# Phenolic Compounds from *Diarthron iranica*: Enzymatic and in Silico Insights Into α-Amylase Inhibitory Activity

**DOI:** 10.5812/ijpr-164807

**Published:** 2025-11-04

**Authors:** Zeinab Yazdiniapour, Reza Gashavi, Tohid Khodabande, Hossein Biganeh, Morteza Sadeghi, Mehran Miroliaei, Mustafa Ghanadian

**Affiliations:** 1 Department of Pharmacognosy, School of Pharmacy and Pharmaceutical Sciences, Isfahan University of Medical Sciences, Isfahan, Iran; 2 Department of Biochemistry, Sa.C., Islamic Azad University, Sanandaj, Iran; 3 Department of Cell and Molecular Biology and Microbiology, Faculty of Biological Science and Technology, University of Isfahan, Isfahan, Iran; 4 Department of Pharmacognosy, Isfahan Pharmaceutical Sciences Research Center, School of Pharmacy and Pharmaceutical Sciences, Isfahan University of Medical Sciences, Isfahan, Iran

**Keywords:** *Diarthron iranica*, Phenolic Compounds, Alpha-Amylase Inhibitors, Coumarins, Lignans

## Abstract

**Background:**

*Diarthron iranica* (family: *Thymelaeaceae*), a medicinal plant native to Iran, contains a variety of beneficial phytochemicals, among which phenolic compounds with a spectrum of health-promoting activities hold a special place.

**Objectives:**

This study deals with the isolation and identification of the main phenolic compounds from *D. iranica* and investigates their inhibitory potential against α-amylase, an important enzyme in glucose metabolism, using in silico and in vitro approaches.

**Methods:**

The purification procedure was accomplished employing chromatographic methods, including thin-layer chromatography (TLC), medium-pressure liquid chromatography (MPLC), and high-performance liquid chromatography (HPLC). The structures were determined using spectroscopic techniques: NMR (^1^H, ^13^C, DEPT), mass spectrometry (MS), and UV-Vis spectroscopy. The in vitro α-amylase inhibition was performed in triplicate across seven concentrations (0.30 - 2.80 mg/mL) using the DNS colorimetric method. Molecular docking simulations were conducted using AutoDock 4.2, with ten conformations generated per ligand.

**Results:**

Several phenolic derivatives, including 5-[(β)-D-xylopyranoside-(1'''→6'')-β-D-glucopyranoside] 7-Methoxy apigenin (yuankanin, 1), 6'-Methoxy-7'-hydroxy-3'-O-7-bicoumarin (daphnoretin, 2), 4,4'-dihydroxy-3,3'-dimethoxy-7, 9’:7’, 9-diepoxylignan known as pinoresinol (3), and kusunokinin (4) with 3',4'-dimethoxy-3,4-methylenedioxydibenzyl butyrolactone structure were isolated and identified. In an α-amylase inhibition assay, compounds 1 and 3 exhibited moderate inhibitory activity with IC_50_ values of 1.32 mg/mL and 1.81 mg/mL, respectively, compared to the reference compound luteolin (IC_50_ = 0.63 mg/mL), indicating effective but relatively weaker inhibition. Compound 2 demonstrated the strongest inhibitory activity with an IC_50_ value of 0.71 mg/mL, surpassing compounds 1 and 3. Molecular docking studies revealed that compound 1 had a superior binding free energy of -7.13 kcal/mol, forming stable interactions through hydrogen bonding and van der Waals forces within the enzyme’s binding site. Compound 3 showed a slightly lower binding energy of -6.43 kcal/mol with fewer stabilizing interactions. However, compound 2 demonstrated poor performance in the docking assay, despite its potent inhibitory activity in the α-amylase assay.

**Conclusions:**

The phytochemical analysis carried out on the aerial parts of *D. iranica* yielded the identification and characterization of four phenolic compounds, including a methoxy apigenin glycoside (1), one bicoumarin (2), and two lignans (3-4). Molecular docking studies indicated that compound 1 exhibited superior inhibitory potential compared to compound 3, with stable interactions in the enzyme’s binding site. In α-amylase inhibitory assays, these compounds displayed varying levels of activity, with compound 2 showing the highest potency (IC_50_ = 0.71 mg/mL), followed by compounds 1 (IC_50_ = 1.32 mg/mL) and 3 (IC_50_ = 1.81 mg/mL). However, all were less effective than the reference compound luteolin (IC_50_ = 0.63 mg/mL), which demonstrated superior efficacy.

## 1. Background

*Diarthron iranica*, also known as *Stelleropsis iranica*, is a member of the Thymelaeaceae family and is native to Western Asia (1). It has smooth green stems and linear leaves that are about 15 - 16 mm long with pointed tips and short petioles. The inflorescence has 5 - 6 flowers with thin, linear sepals and floral structures that are not very noticeable. The plant grows to be 10 to 15 cm tall and blooms with small yellow flowers in the summer (2). Previous phytochemical studies have shown that plants in the Thymelaeaceae family possess a diverse range of bioactive constituents, including phenolics, coumarins, lignans, coumarinolignans, flavonoids, and daphnane-type diterpene esters (3, 4). Among them, flavonoids, lignans, and coumarins are three types of phenolic compounds that have recently attracted considerable attention for their anti-diabetic properties (5, 6). These compounds can reduce blood glucose levels through multiple mechanisms, including activation of signaling pathways such as Akt/PI3K (enhancing insulin function), inhibition of key digestive enzymes like α-amylase and β-glucosidase, as well as limiting glucose absorption from the gastrointestinal tract (7, 8). New studies have explored the inhibitory activity of peptides targeting β-glucosidase, which further highlights the enzyme inhibition in glycemic control. For instance, SAR analysis of these β-glucosidase inhibitory peptides leads to designing new anti-diabetic agents (9).

Certain plant species within this family have demonstrated significant pharmacological efficacy in the management of diabetes mellitus, especially via inhibiting the glucose-metabolizing enzymes (10-12). From an ethnobotanical perspective in Africa, certain plants from the Thymelaeaceae family are recognized as critical therapeutic agents for glycemic control (13), underscoring their cultural and medicinal importance in diabetes management.

The enzyme α-amylase breaks down starch and glycogen into glucose. Phenolics may help lower blood sugar levels by inhibiting this enzyme, which prevents excessive glucose absorption (14). It is thought that these phytochemicals inhibit α-amylase activity by binding directly to the enzyme's active site, which changes its structure in a way that makes it less active (15).

## 2. Objectives

In this study, considering the documented hypoglycemic potential of compounds within the *Thymelaeaceae* family and the endemic distribution of *D. iranica* in Iran, we aim to isolate and characterize its phenolic constituents to evaluate their anti-diabetic properties. Given the current lack of comprehensive in vitro data, this research may help bridge existing scientific gaps regarding the antioxidant functions of lignans and flavonoids and their impact on diabetic-related pathways.

## 3. Methods

### 3.1. General Experimental Procedures

NMR spectra were recorded at 25°C using Bruker 400 MHz spectrometers. Medium-pressure liquid chromatography (MPLC) was carried out using a Buchi 861 apparatus with a silica gel-filled column (15 - 40 μm; 26 mm × 460 mm i.d.). An RP-18 column (36 × 460 mm, LiChroprep^®^ silica gel, Merck, Germany) was used for RP-MPLC. RP-high-performance liquid chromatography (HPLC) analysis was performed on a Waters system equipped with a Shimpack C-18 column (20 × 250 mm, 5 µm; Shimadzu, Japan). Normal phase flash silica gel (40 - 63 μm; Fisher Scientific, Fair Lawn, NJ, USA) was employed for normal-phased column chromatography. Gas chromatography-mass spectrometry (GC-MS) analysis was conducted using an Agilent 7890A gas chromatograph coupled with an Agilent 5975C mass selective detector, equipped with an HP-5 capillary column. Spots were detected using a UV light cabinet and visualized by cerium (IV) sulfate in 2 N sulfuric acid (H_2_SO_4_) solution processed by hair dryer heating. Acetone, dichloromethane, methanol, hexane, ethyl acetate, and all other solvents were purchased from the Dr. Mojallali Chemical Industries Complex (I.R., Iran) and were of analytical grade.

### 3.2. Plant Material

The aerial parts of *D. iranica* were collected in June 2022 from Robat-Sefid, a region located approximately 90 kilometers south of Mashhad, between the cities of Torbat-Heydarieh and Mashhad in Khorasan Razavi province (elevation of 1,700 meters). The species was identified by Mohammad Reza Joharchi, a plant taxonomist, and a herbarium specimen with voucher code (SAM-4233) has been deposited at Samsam-Shariat Herbarium, Department of Pharmacognosy, Isfahan University of Medical Sciences, Iran.

### 3.3. Extraction and Preparation

**Figure 1.**
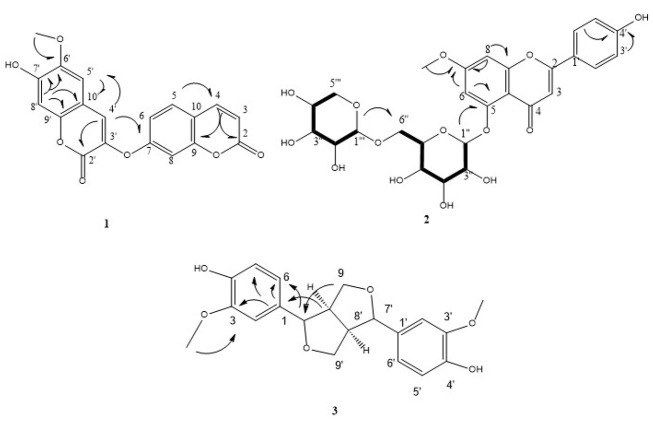
HSQC-TOCSY correlations (bold line), and key HMBC interactions for compounds 1 - 3

**Figure 2.**
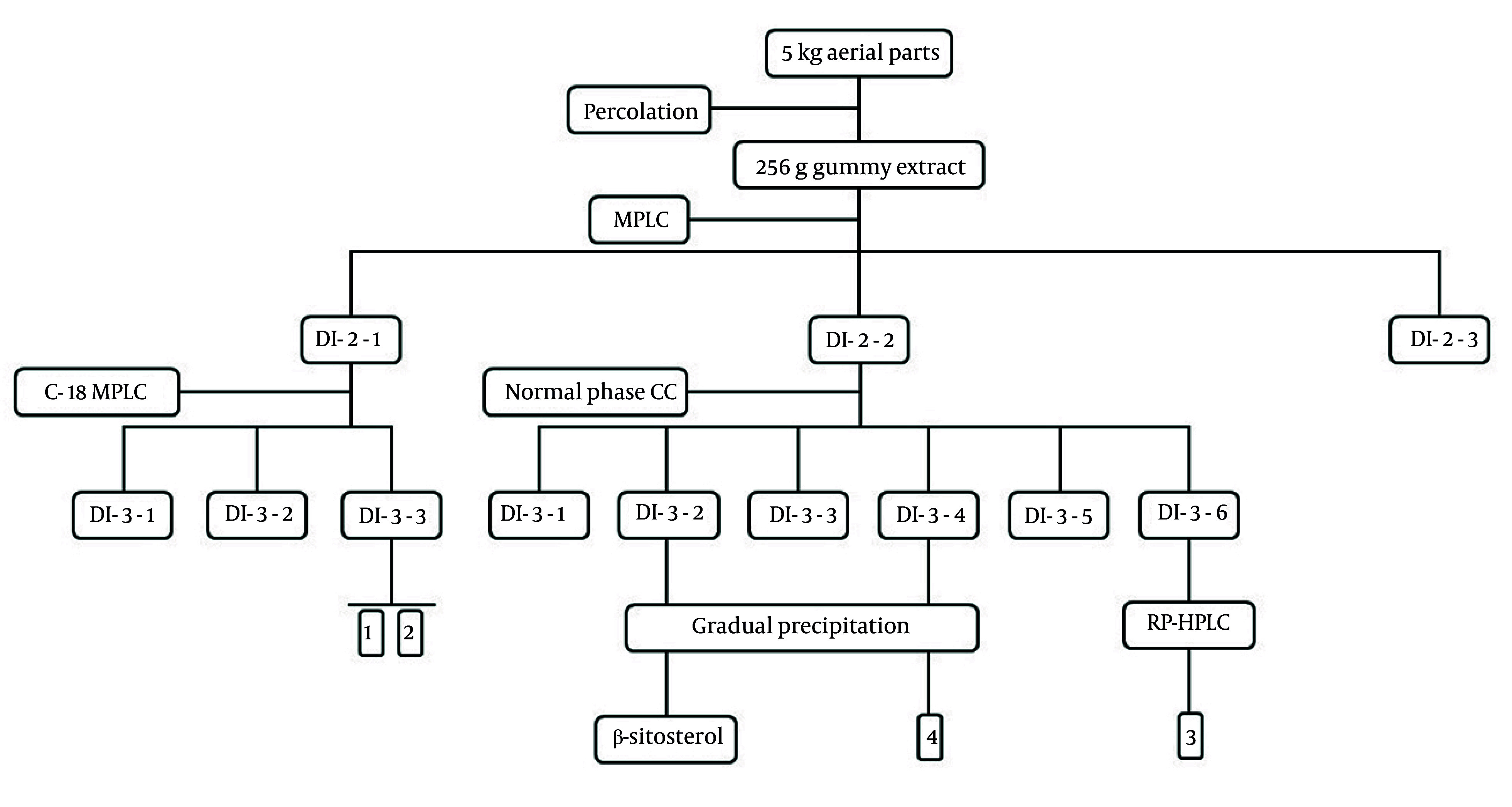
The tree-diagram of extraction and isolation of purified compounds

The percolation method, employing 20 liters of acetone: Dichloromethane (2:1 ratio) over three days, extracted 5 kg of dried powdered plant materials. The extract was then filtered and concentrated using a rotary evaporator, yielding 256 g of gummy extract (DI-1-1). A part of DI-1-1 was applied to the MPLC system containing reversed-phase RP18 using methanol: Water mixtures in sequential gradients (30:70, 2L, DI-2-1; 60:40, 2L, DI-2-2; 90:10, 1L, DI-2-3) as a solvent. Based on thin-layer chromatography (TLC) analysis, fraction DI-2-1 (12 g) underwent further purification on a similar C-18 MPLC setting using elution gradients of MeOH: H_2_O (40:60, 1L, DI-3-1; 50:50, 1L, DI-3-2; 60:40, 1L, DI-3-3), which yielded two pure compounds: Compound 1 (30 mg) and compound 2 (25 mg). Fraction DI-2-2 (26 g), a viscous extract, was coated on celite and applied to a silica gel column (45 - 60 µm, 50 × 3 cm), using a solvent system composed of hexane: Ethyl acetate: Methanol in gradient form (95:5:0, 1.6L, DI-3-1; 90:10:0, 1.6L, DI-3-2; 85:15:0, 1.6L, DI-3-3; 80:20:0, 1.6L, DI-3-4; 75:20:5, 1.6L, DI-3-5, and DI-3-6). The resulting DI-3-2 fraction containing precipitate was initially dissolved in minimal Hex: Acetone, then subjected to gradual precipitation under laboratory conditions. The supernatant was discarded, and the precipitate collected. This process was repeated twice to obtain a pure steroid compound (DI-4-1) identified as β-sitosterol by NMR and mass spectra (16). Fraction DI-3-6 was injected into RP-HPLC using isocratic methanol: Water (6:4) and 0.1 mL trifluoroacetic acid as a mobile phase, to obtain compound 3 (10 mg) in a pure state (Figure 1). Similarly, DI-3-4 was treated with hexane: acetone and precipitated under ambient lab conditions. The repeated purification yielded compound 4 in low quantities, which were submitted to GC-mass analysis for identification (Figure 2). 

### 3.4. 2D and 3D-NMR Experiments

HSQC correlated each proton with its directly bonded carbon. The COSY-DQF experiment allowed the detection of direct connectivities through proton–proton scalar coupling. HMBC allowed partial substructures to be interconnected. The saccharide part and its sugar sequences were identified through HSQC-TOCSY and HMBC experiments. In sugar regions where proton signals are highly overlapped, the HSQC-TOCSY experiment allowed us to resolve individual spin systems by each sugar. It is a 3-dimensional NMR experiment, where the F1 (^1^H)–F2 (^13^C) dimension corresponds to the ^1^H–^13^C HSQC correlations, while the F1 (^1^H)–F3 (^1^H) projection reveals ^1^H–^1^H TOCSY spin system correlations. Connectivities between sugars and with the aglycone part were determined through HMBC spectra. The spectra are provided in the supplementary material file.

### 3.5. α-Amylase Inhibitory Assay

The α-amylase inhibitory activity was evaluated based on the method described by Hua et al. (17), with minor modifications. In brief, 20 μL of α-amylase enzyme (8 U/mL) was mixed with 20 μL of the test compounds at varying concentrations (0.30, 0.42, 0.60, 1.30, 1.80, 2.30, and 2.80 mg/mL) and incubated at 25°C for 10 minutes. The stock solutions were prepared in 0.2 M dimethyl sulfoxide and then diluted in potassium phosphate buffer (200 mM, pH 6.7). Then, 500 μL of starch solution (2%) was added, and the mixture was further incubated for 30 minutes at 25°C. To terminate the reaction, 100 μL of dinitrosalicylic acid reagent was added, and the test tubes were heated at 100°C for 10 minutes. Each experiment was performed in triplicate. After cooling, absorbance was measured at 540 nm. Luteolin was used as a standard drug in this study. Enzyme inhibition percentage was calculated using the following formula: % Inhibition = (A_control_ - A_sample_)/A_control_ × 100.

### 3.6. Molecular Docking

Molecular docking studies were conducted using AutoDock version 4.2 (18) to evaluate the binding interactions between each compound and the 1BAG receptor. Prior to docking, structural optimization of both the receptor and all ligands was carried out utilizing Chimera software version 1.7 (19). Preprocessing steps included the removal of all water molecules from the receptor structure, addition of polar hydrogens, and assignment of Kollman partial charges. Subsequently, the docking grid box was defined with dimensions set to 75 × 75 × 75 points and a spacing of 0.368 Å. For each ligand, ten binding conformations were generated, and the conformation corresponding to the lowest binding energy was selected for further analysis. Visualization and analysis of ligand-receptor interactions were subsequently performed using Discovery Studio Visualizer version 16.2.0.16349 (20), which facilitated the generation of two-dimensional representations of the final ligand-protein complexes.

### 3.7. Statistical Analysis

Results are presented as mean ± standard error of the mean (SEM). Data analysis and interpretation were performed using GraphPad Prism and Microsoft Excel software.

## 4. Results

Phytochemical analysis of *D. iranica* yielded four phenolic derivatives, including one flavonoid glycoside, one bicoumarin, and two lignans, characterized by the following spectral data.

### 4.1. Spectral Data of Isolated Compounds

- Compound 1:

^1^H-NMR (400 MHz, DMSO-d6): δ 2.99 (^1^H, m, H-2″), 3.02 (^1^H, m, H-5‴), 3.11 (^1^H, m, H-3″), 3.21 (^1^H, m, H-4‴), 3.28 (^1^H, m, H-4″), 3.36 (^1^H, m, H-2‴), 3.57 (^1^H, m, H-3‴), 3.64 (^1^H, bd, J = 10.7 Hz, H-6″), 3.66 (^1^H, m, H-5″), 3.68 (^1^H, m, H-5‴), 3.90 (^3^H, s, H-OMe-7), 3.97 (^1^H, bd, J = 10.7 Hz, H-6″), 4.19 (^1^H, d, J = 7.5 Hz, H-Xyl-1‴), 4.78 (^1^H, d, J = 7.5 Hz, H-GLC-1″), 6.70 (^1^H, s, H-3), 6.87 (^1^H, d, J = 2.4 Hz, H-6), 6.91 (^2^H, d, J = 8.8 Hz, H-3′, H-5′), 7.04 (^1^H, d, J = 2.4 Hz, H-8), 7.94 (^2^H, d, J = 8.8 Hz, H-2′, H-6′). ^13^C-NMR (100 MHz, DMSO-d6): δ 56.13 (C-OMe-7), 65.67 (C-5‴), 68.69 (C-6″), 69.54 (C-4″), 69.78 (C-4‴), 73.39 (C-2″), 73.49 (C-2‴), 75.96 (C-5″, C-3‴), 76.59 (C-3″), 96.67 (C-8), 103.00 (C-6), 103.83 (C-GLC-1″), 104.13 (C-Xyl-1‴), 105.66 (C-3), 109.18 (C-10), 116.05 (C-3′, C-5′), 120.81 (C-1′), 128.17 (C-2′, C-6′), 158.13 (C-5), 158.42 (C-9), 161.45 (C-4′), 161.46 (C-2), 163.58 (C-7), and 176.86 (C-4); negative ESIMS at m/z 579.1693 [M - H]^-^.

- Compound 2:

^1^H-NMR (400 MHz, DMSO-d6): δ 3.81 (^3^H, s, H-6-OMe), 6.37 (^1^H, d, J = 9.5 Hz, H-3), 6.83 (^1^H, s, H-8′), 7.10 (^1^H, dd, J = 8.6, 2.5 Hz, H-6), 7.17 (^1^H, d, J = 2.4 Hz, H-8), 7.19 (^1^H, s, H-5′), 7.70 (^1^H, d, J = 8.6 Hz, H-5), 7.86 (^1^H, s, H-4′), 8.04 (^1^H, d, J = 9.6 Hz, H-4). ^13^C-NMR (100 MHz, DMSO-d6): δ 56.44 (C-6-OMe), 103.20 (C-8′), 104.41 (C-8), 109.71 (C-5′), 113.85 (C-6), 114.31 (C-3), 114.81 (C-10), 130.35 (C-5), 131.52 (C-4′), 135.81 (C-3′), 144.53 (C-4), 146.36 (C-6′), 148.12 (C-9′), 151.60 (C-7′), 155.46 (C-9), 157.48 (C-2′), 160.20 (C-2), and 160.44 (C-7); negative ESIMS at m/z 351.0552 [M - H]^-^.

- Compound 3:

^1^H-NMR (400 MHz, CDCl_3_): δ 6.86 (^2^H, s, H2 and H2′), 6.70 (^2^H, d, J = 5.6 Hz, H5 and H5′), 6.72 (^2^H, dd, J = 4.1, 5.6 Hz, H6 and H6′), 4.59 (^2^H, d, J = 2.3 Hz, H7 and H7′), 3.00 (^2^H, m, H8 and H8′), 3.70 (^2^H, dd, J = 2.3, 5.7 Hz, H9a and H9a′), 4.11 (^2^H, dd, J = 0.5, 5.7 Hz, H9b and H9b′), 3.74 (^3^H, s, 3-OMe and 3′-OMe). ^13^C-NMR (100 MHz, CDCl_3_): δ 55.15 (C8, C8′), 56.42 (3-OMe, 3′-OMe), 72.49 (C9, C9′), 87.31 (C7, C7′), 110.94 (C2, C2′), 116.11 (C5, C5′), 120.01 (C6, C6′), 133.74 (C1, C1′), 147.10 (C4, C4′), and 148.99 (C3, C3′); negative ESIMS at m/z 357.1338 [M - H]^-^.

**Figure 3.**
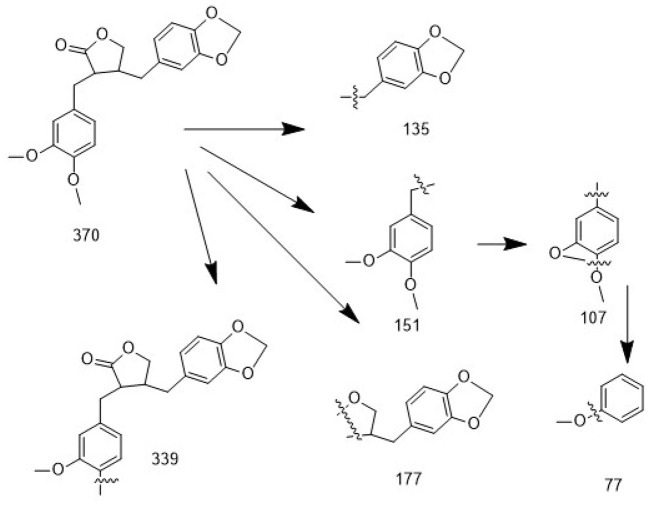
Fragmentation pattern of the mass spectrum for compound 4: In the fragmentation analysis of the mass spectrum, the presence of fragment ions at m/z 339, 177, 151, 135, 107, and 77 provides structural confirmation of the compound as kusunokinin.

- Compound 4 was structurally identified as kusunokinin with a 3',4'-dimethoxy-3,4-methylenedioxy dibenzyl butyrolactone structure, using GC-MS (Figure 3). 

### 4.2. α-Amylase Inhibitory Activity

**Figure 4.**
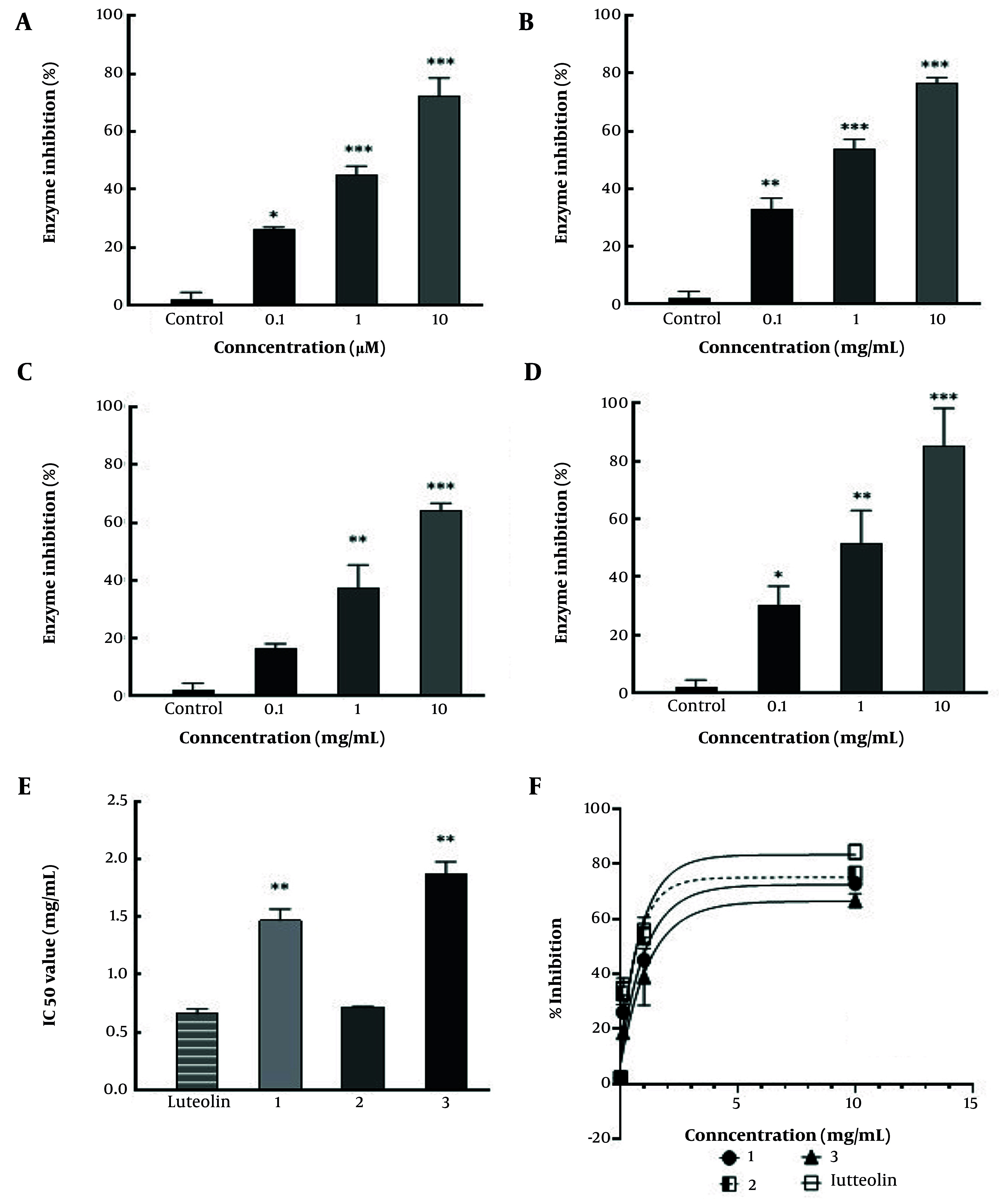
A, Comparative α-amylase inhibitory activity of compound 1; B, 2, and C, 3 D, versus luteolin ; E, IC_50_ values of compounds 1 - 3 versus luteolin as control – the graph illustrates the dose-dependent inhibition profiles of all three samples across various concentrations (0.1 - 10 mg/mL); F, dose-response curves for inhibition of α-amylase by compounds 1 - 3 and luteolin. The X-axis shows log [concentration, mg/mL], and the Y-axis shows % inhibition (* P < 0.5, ** P < 0.01, and *** P < 0.001 compared to control).

The α-amylase inhibitory activity of compounds 1 - 3 was evaluated in comparison with luteolin, used as the reference standard. At a ligand concentration of 0.1 mg/mL, compound 1 showed 26.1% enzyme inhibition, which increased to 72.46% inhibition at 10 mg/mL. The IC_50_ value for compound 1 was calculated to be 1.32 mg/mL (R^2^ = 0.93, RMSE = 9.58). Compound 2 exhibited 32.8% inhibition at 0.1 mg/mL, which increased to 76.6% at 10 mg/mL. Its IC_50_ was determined to be 0.71 mg/mL (R^2^ = 0.90, RMSE = 12.38). Compound 3 showed 17.6% inhibition at 0.1 mg/mL and 68.5% inhibition at 10 mg/mL, with an IC_50_ value of 1.81 mg/mL (R^2^ = 0.95, RMSE = 8.25). Luteolin, serving as the standard compound, demonstrated 29.1% inhibition at 0.1 mg/mL and reached 87.5% inhibition at 10 mg/mL, with an IC_50_ of 0.63 mg/mL as determined from the dose-response curve (R^2^ = 0.89, RMSE = 13.59) (18, 20-23). Luteolin exhibited the highest α-amylase inhibition, while compound 2 demonstrated strong inhibitory activity with an IC_50_ value of 0.71 mg/mL, followed by compounds 1 and 3 with moderate inhibition, showing IC_50_ values of 1.32 mg/mL and 1.81 mg/mL, respectively, compared to luteolin’s IC_50_ of 0.63 mg/mL (Figure 4). 

### 4.3. Molecular Docking Evaluation

**Figure 5.**
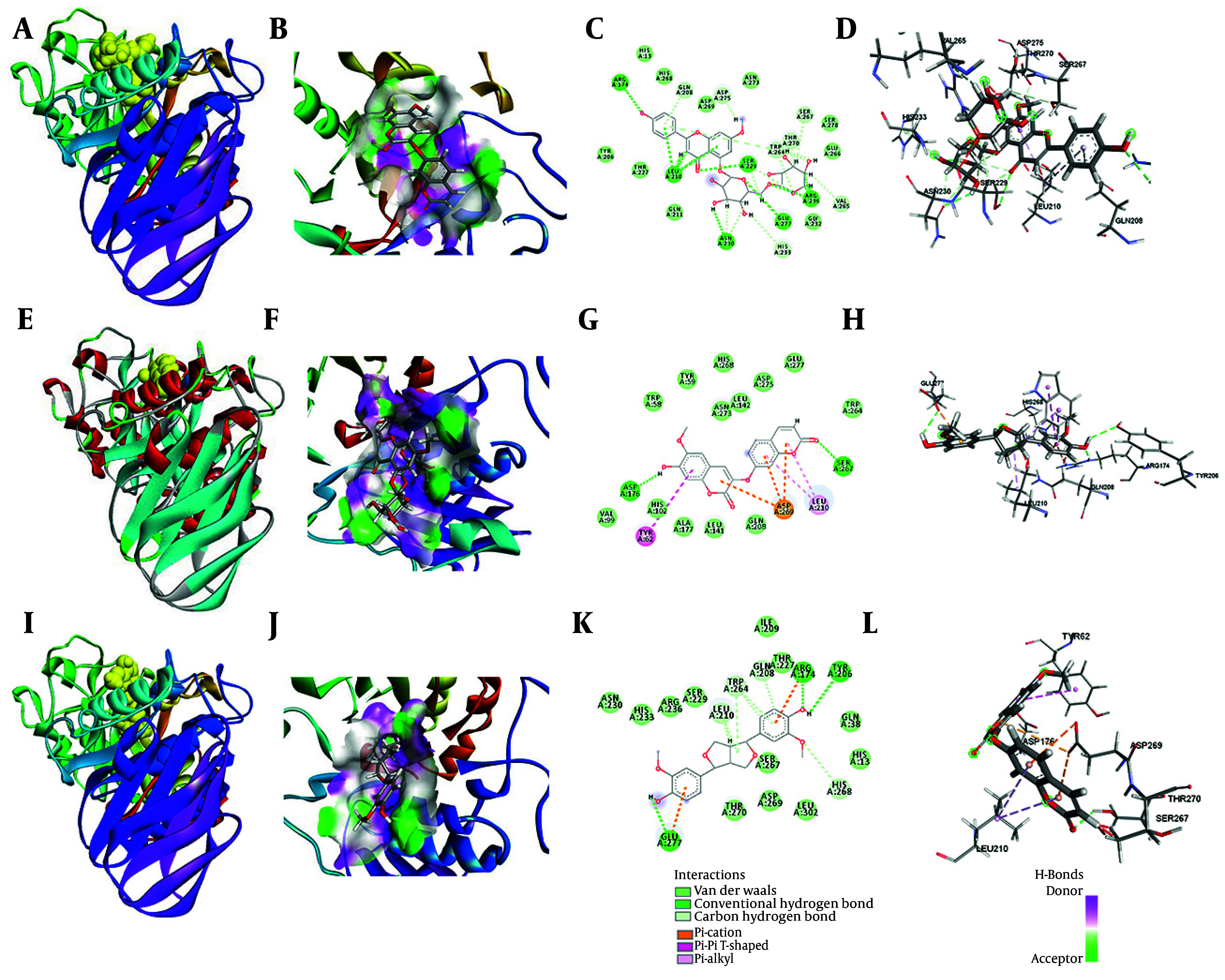
A, overall structure of the compound 1-α-amylase complex as visualized in the docking simulations; B, hydrophobic surface of interaction between compound 1 and α-amylase; C, 2D interaction map of the compound 1-α-amylase complex; D, 3D visualization of the binding interactions between compound 1 and α-amylase; E, overall structure of the compound 2-α-amylase complex as obtained from the docking simulations; F, hydrophobic surface of interaction between compound 2 and α-amylase; G, 2D interaction map of the compound 3-α-amylase complex; H, 3D visualization of the binding interactions between compound 2 and α-amylase; I, overall structure of the compound 3-α-amylase complex as obtained from the docking simulations; J, hydrophobic surface of interaction between compound 3 and α-amylase; K, 2D interaction map of the compound 3-α-amylase complex; L, 3D visualization of the binding interactions between compound 3 and α-amylase.

The interactions between selected phenolic compounds and α-amylase were investigated using molecular docking techniques (21, 22). The docking simulations produced multiple binding poses for each compound, and the highest-ranked conformations' binding affinities were calculated. Table 1 summarizes the estimated affinities of all ligands examined towards α-amylase and provides details on the resulting binding energies and interaction profiles. As expected, luteolin, the positive control, exhibited a binding energy of -7.97 kcal/mol with α-amylase, supporting its well-known inhibitory properties. Among the tested phenolics, compound 1 demonstrated a superior binding affinity compared to the other metabolites when interacting with the target receptors (-7.13 kcal/mol). A detailed analysis of the interactions found nine hydrogen bonds formed between compound 1 and the residues Arg236, Glu277, Ser229, Asn230, Leu210, and Arg174 in the active site of the enzyme. Additionally, several carbon–hydrogen bonds were observed with Ser278, Glu266, Asn273, Asp269, and His268. Fifteen van der Waals interactions, mostly between Ser267, Thr270, Trp264, Gly232, Gln211, His13, and His268, made the complex even more stable. Most of the residues that took part in these interactions are found in the catalytic domain of α-amylase, as shown in Figure 5. 

**Table 1.** The Docking Energy (kcal/mol) of the Possible α-Amylase Inhibitors

Compounds	Docking Energy	Van der Waals	Electrostatics	Hydrogen Bond	Ligand Efficiency
**Compound 1**	-7.13	-4.55	-0.81	-1.37	-0.41
**Compound 2**	-6.21	-3.41	-0.75	-1.96	-0.09
**Compound 3**	-6.43	-4.11	-0.72	-1.15	-0.45
**Luteolin (Ref.)**	-7.97	-5.33	-0.66	-1.68	-0.34

For compound 2, the binding energy determined for the compound 2-α-amylase complex was -6.21 kcal/mol, also denoting a favorable and stable interaction. Interaction profiling identified two conventional hydrogen bonds between compound 2 and amino acid residues Ser267 and Asp176. Additionally, Pi-Anion bond and Pi-shaped interactions were observed with Asp269 and Tyr62, respectively (Figure 5B, E, and H).

For compound 3, the binding energy of the compound 3-α-amylase complex was -6.43 kcal/mol, indicating a favorable and stable interaction. Interaction profiling identified nine conventional hydrogen bonds between compound 3 and amino acid residues Tyr206, Arg174, and Glu277. Carbon-hydrogen bonds were detected with His268, Leu210, and Trp264, and eleven van der Waals interactions were mapped as contributing to the stabilization of the complex. The engaged residues for compound 3 were similarly localized predominantly within the catalytic pocket (Figure 5C, F, and I).

## 5. Discussion

*Diarthron iranica* was phytochemically analyzed employing various chromatographic methods, which isolated four phenolic derivatives, including one 7-methoxy apigenin glycoside (1), one bicoumarin (2), and two lignans (3-4). Compound 1 was purified by solvent washing to eliminate minor impurities from flaky crystalline deposits formed along the inner wall of the reaction tube. The residue underwent multiple recrystallization steps, and the final product was washed with methanol on filter paper. This procedure yielded a white solid agent that exhibited a positive result with ferric chloride and the natural product reagents. In the ^1^H-NMR spectrum, a spin system was observed at δH 6.87 (d, J = 2.4 Hz, 1H) and δH 7.04 (d, J = 2.4 Hz, 1H), associated with two meta-coupled protons. A singlet at δH 6.70 (s, 1H) and an AA′BB′ spin system at δH 7.94 (d, J = 8.8 Hz, 2H) and δH 6.91 (d, J = 8.8 Hz, 2H) indicated a flavone-like structure, presumably related to apigenin. A methoxy group was detected at δH 3.90 (s, 3H), and signals between δH 3.00 - 5.50 suggested the presence of two sugar moieties. ^13^C-NMR analysis revealed signals at δC 103.83, 73.39, 76.59, 69.54, 75.96, and 68.69, consistent with glucose connected via the C-6 position. Additional signals at δC 104.13, 73.49, 75.96, 69.78, and 65.67 were attributed to xylose (23). Overlapping signals and mapping of intra-sugar correlations were resolved using the HSQC-TOCSY spectrum. Further analysis via COSY-DQF enabled identification of direct proton-proton couplings. Finally, HMBC correlations confirmed the attachment sites of the sugar units and the methoxy group to the flavone core (Figure 1). The final structure was elucidated as 5-[(β-D-xylopyranoside-(1″→6″)-β-D-glucopyranoside]-7-methoxy-apigenin, known as yuankanin, which has been previously isolated from Daphne odora Thunb. var. marginata (24).

Compound 2 was similarly purified by solvent washing of reddish-brown crystalline deposits formed on the inner wall of the tube. A small amount of solvent was used to remove small impurities, and then the process of recrystallization was repeated. The final sample was washed with cold methanol on filter paper, producing a pale orange solid that also reacted favorably with ferric chloride. A vinyl proton spin system, characteristic of a coumarin structure, was detected in the ^1^H-NMR spectrum at δH 6.36 (d, J = 9.5 Hz) and δH 8.02 (d, J = 9.6 Hz). The aromatic ring of the coumarin moiety was represented by an ABX spin system that emerged at δH 7.17 (d, J = 2.5 Hz, A of ABX), δH 7.10 (dd, J = 2.5, 8.6 Hz, B of ABX), and δH 7.69 (d, J = 8.6 Hz, X of ABX). An aryl methoxy group was also responsible for the detection of two alkenic singlets at δH 6.83 (bs) and δH 7.86 (s), a phenolic hydroxyl proton at δH 7.86 (s), and a singlet at δH 3.76 (s, 3H). ^13^C-NMR analysis indicated that the compound is a bis-coumarin derivative, featuring two coumarin units linked via an ether bridge. HMBC correlations confirmed the bis-coumarin framework and the methoxy substitution site. Based on HMBC data and spectral similarities to known bis-coumarin derivatives, the structure was proposed as 6′-methoxy-7′-hydroxy-3′-O-7-bicumarin, commonly known as daphnoretin (Figure 1). NMR data were compared with reference values from the whole aerial parts of Streptocaulon griffithii (23).

Compound 3 was inferred to have a molecular formula of C_20_H_22_O_6_, consistent with the DEPT and ^13^C-NMR spectral data and the calculated hydrogen count. Two methoxy groups were detected at δC 42.56 (3′-OMe and 3-OMe) in both the ^13^C-NMR and DEPT spectra, along with two oxygenated methylene carbons at δC 49.72 (C9′ and C9). Six olefinic methine carbons were observed at δC 110.94 (C2′ and C2), 116.11 (C5′ and C5), and 120.01 (C6′ and C6). Additionally, two simple aliphatic carbons appeared at δC 55.15 (C8 and C8′), and two oxygenated aliphatic methine carbons at δC 87.13 (C7 and C7′). Six quaternary carbons were identified: Two non-oxygenated olefinic carbons at δC 133.74 (C1′ and C1), and four oxygenated olefinic carbons at δC 148.99 (C3′ and C3) and 147.10 (C4′ and C4). Based on the carbon environments, the following proton types were deduced: Six protons from two methoxy groups (–OCH_3_), four from two oxygenated methylene groups (–CH_2_–O), six from olefinic methine (=CH–), two from aliphatic methine (–CH–), and two from oxygenated aliphatic methine carbons — totaling 20 protons. The remaining two protons, not accounted for in the DEPT and ^13^C-NMR spectra, are attributed to two hydroxyl groups (–OH) from phenolic moieties. These signals support the molecular formula C^20^H^22^O^6^, and correspond to two benzene rings substituted at positions 1, 3, and 4. Altogether, the spectral data support the structure of 3′,3-dimethoxy dibenzyl butyrolactone, confirming the identity of the compound as 4,4'-dihydroxy-3,3'-dimethoxy-7, 9’:7’, 9-diepoxylignan known as pinoresinol (Figure 1) (25).

For compound 4, the analysis was performed by comparing the fragmentation pattern with reference spectra available in the NIST mass spectral database, confirming the proposed structure. Based on the mass spectrum and the match factor (MF) parameter, the degree of similarity between the identified compound’s spectrum and the reference spectrum in the library was assessed. Higher MF values (closer to 1000) indicate stronger similarity, meaning that most peaks in the two spectra closely correspond. An MF score of 902 for compound 4 reflects a very strong match with kusunokinin mass spectra. Moreover, fragmentation analysis of the mass spectrum revealed key ions at m/z 135, 151, 107, 339, 177, and 77, which strongly support the proposed structure and align with characteristic cleavage pathways consistent with previously reported data (Figure 3) (26). The physicochemical properties of isolated compounds (1 - 4) are summarized in Table 2. 

**Table 2.** Physicochemical Properties of Isolated Compounds

Compounds	Molecular Formula	MW(g/mol)	State
**1**	C_27_H_30_O_14_	578.5	White solid powder
**2**	C_19_H_12_O_7_	352.3	Reddish-brown crystall
**3**	C_20_H_22_O_6_	358.4	White solid
**4**	C_21_H_22_O_6_	370.4	White solid

Considering the α-amylase inhibitory study, luteolin at equivalent concentrations exhibited a higher inhibition rate (29.1% inhibition at 0.1 mg/mL and 87.5% at 10 mg/mL) compared to the phenolics. Both compounds 1 and 3 demonstrated moderate, dose-dependent inhibition, with IC_50_ values of 1.32 mg/mL and 1.81 mg/mL, respectively, whereas compound 2 showed stronger activity, achieving an IC_50_ of 0.71 mg/mL. The bioassay results of isolated compounds are in agreement with the literature. These findings are consistent with previous reports. In a study by Li et al. (10), which investigated phytochemicals from *Edgeworthia gardneri* (Wall.) Meisn. effective in suppressing glycemic enzymes, compound 2 demonstrated potent activity against α-amylase and α-glucosidase, with IC_50_ values of 121.7 ± 2.9 µM and 183.5 ± 2.3 µM, respectively. Similarly, findings from an in vitro study showed that, among the coumarins isolated from the flowers of *E. gardneri*, compound 2 was the most potent, suppressing both enzymes with IC_50_ values of 90.0 ± 4.1 µg/mL and 86.0 ± 3.0 µg/mL for α-amylase and α-glucosidase, respectively (27). Therefore, based on the findings of the present study and previous research, this compound may be considered a lead candidate for further investigation.

Besides, considering flavonoids' hypoglycemic function, a systematic review done by Lamet al. exhibited the potential of flavonoids as α-amylase inhibitors for managing diabetes by analyzing in vitro studies (28). Furthermore, a recent study reviewing the molecular mechanisms underlying the hypoglycemic effects of flavonoids demonstrated that they modulate glycemic targets and signaling pathways, particularly enzymes involved in carbohydrate metabolism, such as α-amylase and α-glucosidase (29). However, to the best of our knowledge, no studies are available about the amylase or glucosidase inhibitory potential of compound 1, but extensive research has been conducted on its apigenin skeleton and derivatives to evaluate their inhibitory effects on metabolizing enzymes. Concerning this, among the 40 flavone structures analyzed for α-amylase and α-glucosidase, apigenin appeared in 30 studies, highlighting its relevance in enzyme inhibition research. Despite varied substitutions, particularly hydroxy and methoxy groups across rings A, B, and C, apigenin remains a candidate for α-amylase inhibition for antidiabetic applications (28). In another study conducted by Sadeghi et al. (as cited by Dadkhah et al.), researchers investigated the anti-glycation potential of apigenin and its glycosidic derivatives, namely apigenin-4'-O-glucoside (A4′G) and apigenin-7-O-glucoside (A7G), in ribose-induced glycation of human serum albumin (HSA). The results showed that both derivatives, particularly A4′G, significantly reduce advanced glycation end products (AGEs) and “cross-β structures” associated with protein damage (30).

On the other hand, lignans like pinoresinol and its derivatives have garnered significant interest in the setting of metabolic syndrome disorders, particularly in managing hyperglycemia (31, 32). In a preclinical study by Youssef et al. (33), it was revealed that pinoresinol-4-O-β-D-glucopyranoside isolated from Prunus domestica showed prominent antihyperglycemic activity both in vitro and in vivo. However, it showed weak α-amylase activity, strongly inhibited α-glucosidase function, and caused a significant decrease in serum glucose level in the streptozotocin-treated mouse model of diabetes. Another study on phenolics isolated from olive mill wastes exhibited the superior activity of 1-acetoxypinoresinol compared to pinoresinol against α-amylase and α-glucosidase enzymes’ activity (34). Further, pinoresinol isolated from the Fruits of *Terminalia boivinii* revealed stronger inhibitory effects on α-glucosidase compared to acarbose but demonstrated lower efficacy in inhibiting α-amylase and lipase (35). The aforementioned results, which are consistent with our study, confirm the limited α-amylase inhibitory activity of pinoresinol.

However, the findings from the docking simulations suggest that compound 1 possesses a markedly higher binding affinity for α-amylase compared to compounds 2 and 3, as evidenced by its more negative minimum docking energy. This enhanced affinity is likely attributable to the greater number and diversity of non-covalent interactions, particularly the higher incidence of hydrogen bonds and van der Waals contacts, formed within the enzyme’s active site. The spatial clustering of interacting residues within the catalytic domain further implies that both compounds preferentially target and stabilize the biologically relevant active site region of α-amylase. The presence of multiple types of non-covalent interactions, including conventional hydrogen bonds and van der Waals forces, significantly contributes to the overall conformational stability and binding affinity of the complexes. The superior binding profile of compound 1 highlights its potential as a highly effective molecular inhibitor of α-amylase activity, while compound 2 also demonstrated notable inhibitory interactions. Collectively, these data underscore the potential of both compounds as α-amylase inhibitors, with compound 1 offering a more favorable interaction landscape. This provides a structural rationale for prioritizing compound 1 in future investigations, while recognizing compound 2 as a promising complementary candidate for α-amylase–targeted therapeutic exploration.

### 5.1. Conclusions

The phytochemical analysis of *D. iranica* aerial parts led to the isolation and identification of four phenolics, namely yuankanin, daphnoretin, pinoresinol, and kusunokinin. Considering α-amylase inhibitory activity, luteolin at equivalent concentrations unveiled comparable activity with compound 2 but a stronger effect than 1 and 3. Compounds 1, 2, and 3 displayed inhibitory activity with IC_50_ values of 1.32 mg/mL, 0.71 mg/mL, and 1.8 mg/mL, respectively. However, molecular docking results indicated that compound 1 binds more strongly to the α-amylase active site than compounds 2 and 3, due to a greater number of non-covalent interactions, particularly hydrogen bonds and van der Waals forces. These findings suggest that compound 1 represents a promising candidate for further therapeutic development targeting α-amylase, while compound 2 also demonstrates notable inhibitory potential.

ijpr-24-1-164807-s001.pdf

## Data Availability

The data presented in this study are uploaded during submission and are openly available for readers upon request.
