# Supplementary material for: Phenolic Compounds from Diarthron iranica: Enzymatic and in Silico Insights Into α-Amylase Inhibitory Activity
Source: Iran J Pharm Res. 2025 Nov 4;24(1):e164807. doi: 10.5812/ijpr-164807 (PMC12606866; doi:10.5812/ijpr-164807)
Supplement: ijpr-24-1-164807-s001.pdf [file ijpr-24-1-164807-s001.pdf]

*Supplementary materials of the article*

# Phenolic compounds from *Diarthron iranica*: Enzymatic and In Silico Insights into α-amylase Inhibitory Activity

Zeinab Yazdiniapour[1], Reza Gashavi[2], Tohid Khodabaneh[3], Hussein Biganeh[4], Morteza Sadeghi[5], Mehran Miroliaei[6], Mustafa Ghannadian[7*]

[1]*Department of Pharmacognosy, School of Pharmacy and Pharmaceutical Sciences, Isfahan University of Medical Sciences, Isfahan, Iran; z.yazdianpour@gmail.com*

[2]*Department of Pharmacognosy, School of Pharmacy and Pharmaceutical Sciences, Isfahan University of Medical Sciences, Isfahan, Iran*

[3]*Department of Pharmacognosy, School of Pharmacy and Pharmaceutical Sciences, Isfahan University of Medical Sciences, Isfahan, Iran*

[4]*Department of Pharmacognosy, School of Pharmacy and Pharmaceutical Sciences, Isfahan University of Medical Sciences, Isfahan, Iran. https://orcid.org/0000-0002-2730-5029; biganeh75@gmail.com*

[5]*Department of Biochemistry, Sa.C., Islamic Azad University, Sanandaj, Iran, https://orcid.org/0000-0002-5027-4777; ms.biochemistry2015@gmail.com*

[6]*Department of Cell and Molecular Biology & Microbiology, Faculty of Biological Science and Technology, University of Isfahan, Isfahan, Iran; https://orcid.org/0000-0003-0916-3757; mmiroliaei@yahoo.com*

[*7]*Department of Phytochemistry, Isfahan Pharmaceutical Sciences Research Center, School of Pharmacy and Pharmaceutical Sciences, Isfahan University of Medical Sciences, Isfahan, Iran; https://orcid.org/0000-0001-6446-4734; ghannadian@gmail.com*

## Contents

Fig.S 1.$^{1}$H NMR (400.15 MHz) spectrum of compound **1** ........ 3

Fig.S 2.$^{13}$C NMR (100.6 MHz) spectrum of compound **1** ........ 3

Fig.S 3. DEPT-135 spectrum of compound **1** ........ 4

Fig.S 4. HSQC spectrum of compound **1** ........ 5

Fig.S 5. HMBC spectrum of compound **1** ........ 5

Fig.S 6.H-H TOCSY spectrum of compound **1** ........ 6

Fig.S 7. HSQC-TOCSY spectrum of compound **1** ........ 6
Fig.S 8. DQF-COSY spectrum of compound **1** ........ 7
Fig.S 9. ESIMS spectrum of compound 1 m/z 579.1693[M - H]$^-$. ........ 7
Fig.S 10.$^1$H NMR (400.15 MHz) spectrum of compound **2** ........ 8
Fig.S 11.$^{13}$C NMR (100.6 MHz) spectrum of compound **2** ........ 8
Fig.S 12. DEPT-135 spectrum of compound **2** ........ 9
Fig.S 13.HSQC spectrum of compound **2** ........ 9
Fig.S 14.HMBC spectrum of compound **2** ........ 9
Fig.S 15. ESIMS spectrum of compound **2** m/z 351.0552[M - H]$^-$. ........ 10
Fig.S 16.$^1$H NMR (400.15 MHz) spectrum of compound **3** ........ 10
Fig.S 17.$^{13}$C NMR (100.6 MHz) spectrum of compound **3** ........ 11
Fig.S 18.DEPT-135 spectrum of compound **3** ........ 12
Fig.S 19. DEPT-90 spectrum of compound **3** ........ 12
Fig.S 20. HSQC spectrum of compound **3** ........ 13
Fig.S 21. ESIMS spectrum of compound **3** m/z 357.1338[M - H]$^-$. ........ 13

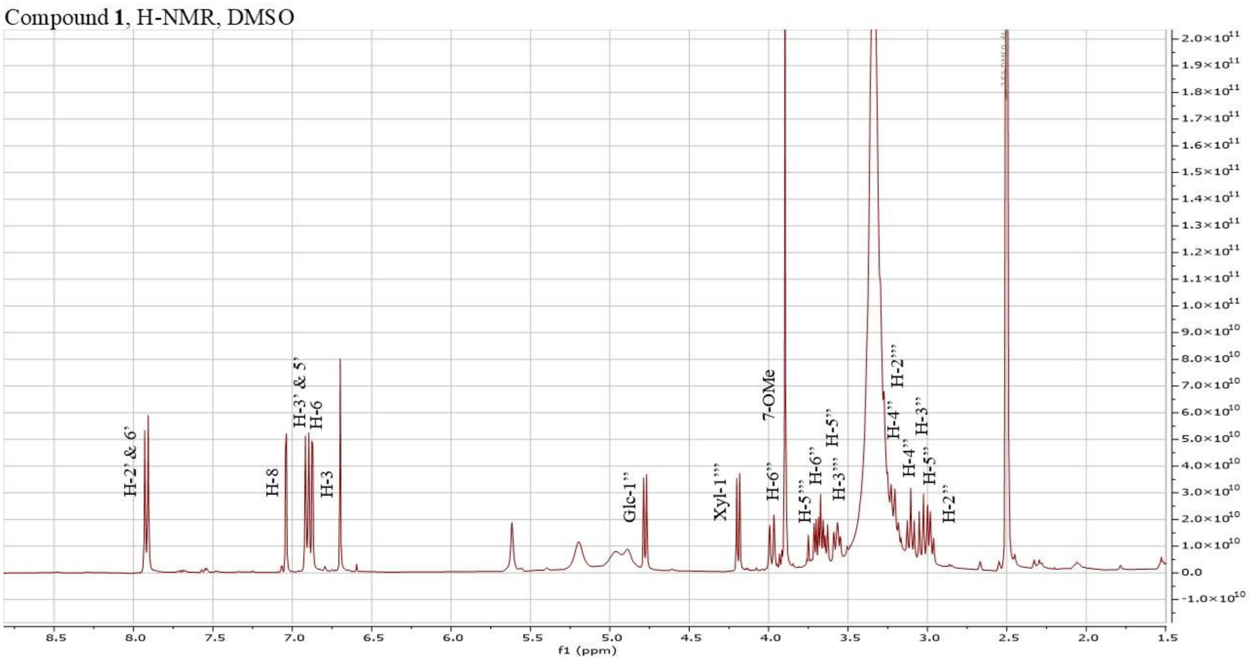


Fig.S 1.$^{1}$H NMR (400.15 MHz) spectrum of compound **1**

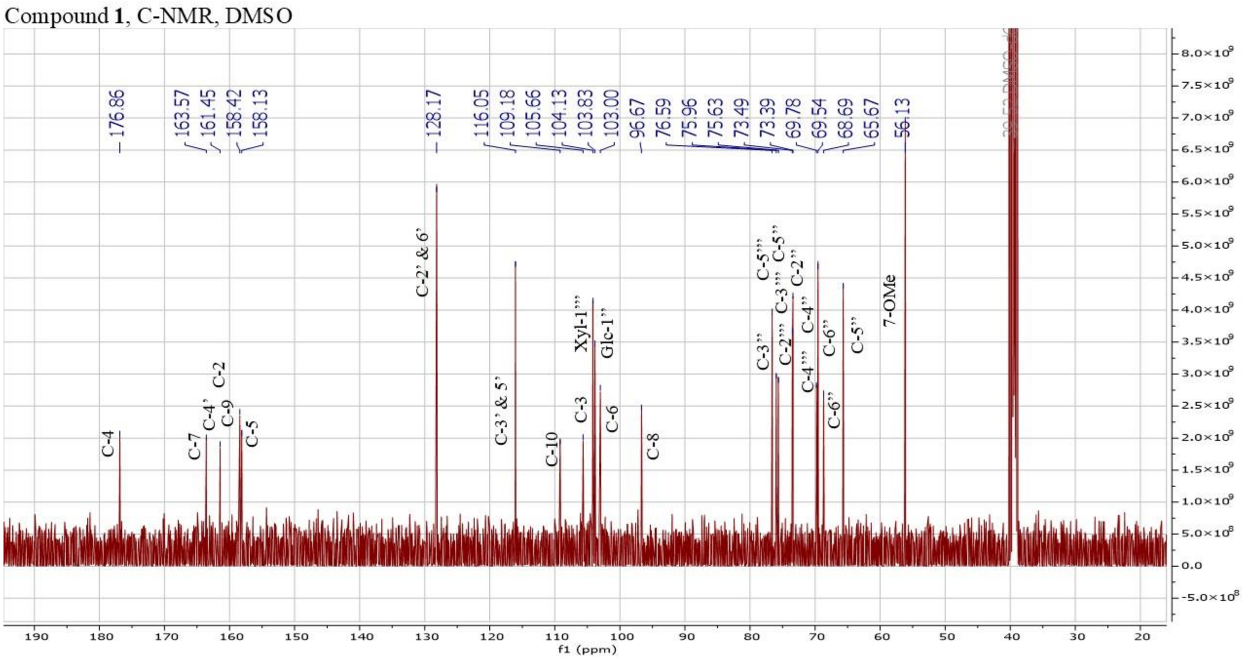


Fig.S 2.$^{13}$C NMR (100.6 MHz) spectrum of compound **1**

Compound **1**, DEPT-135, DMSO

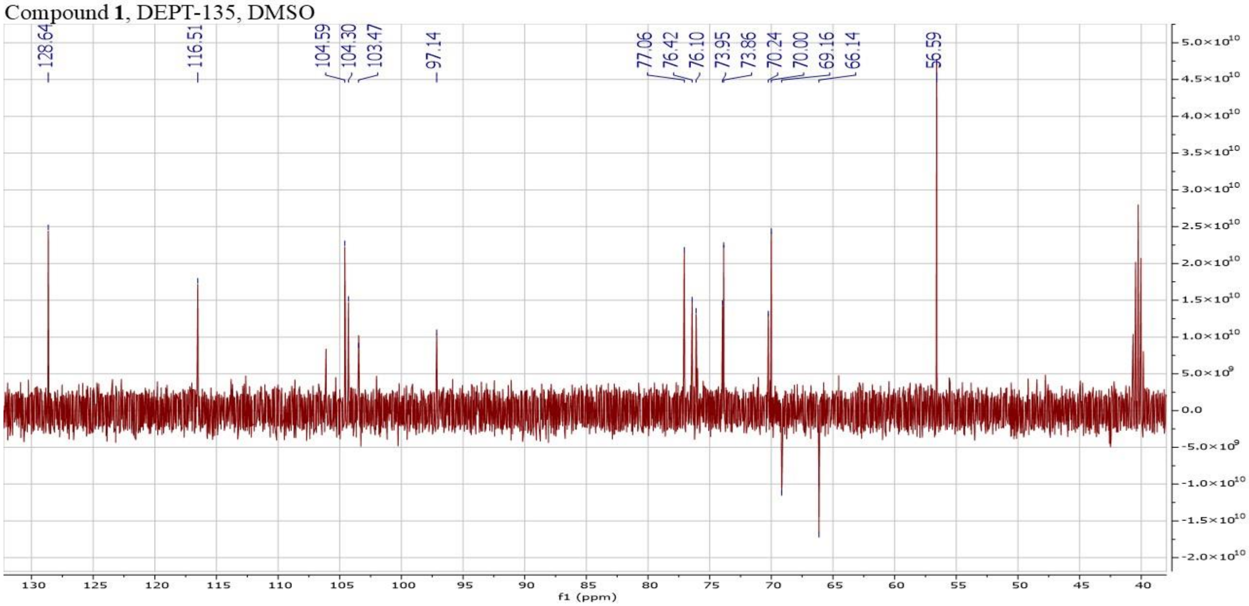


Fig.S 3. DEPT-135 spectrum of compound **1**

Compound **1**, HSQC, DMSO

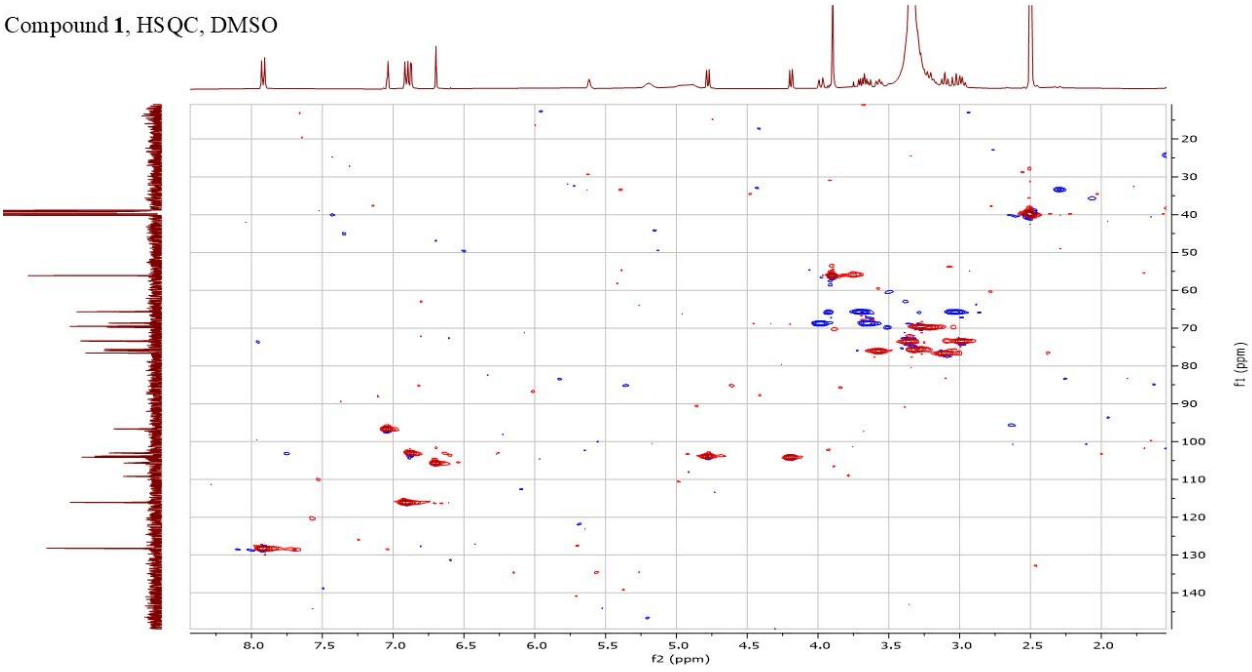

Fig.S 4. HSQC spectrum of compound **1**

Compound **1**, HMBC, DMSO

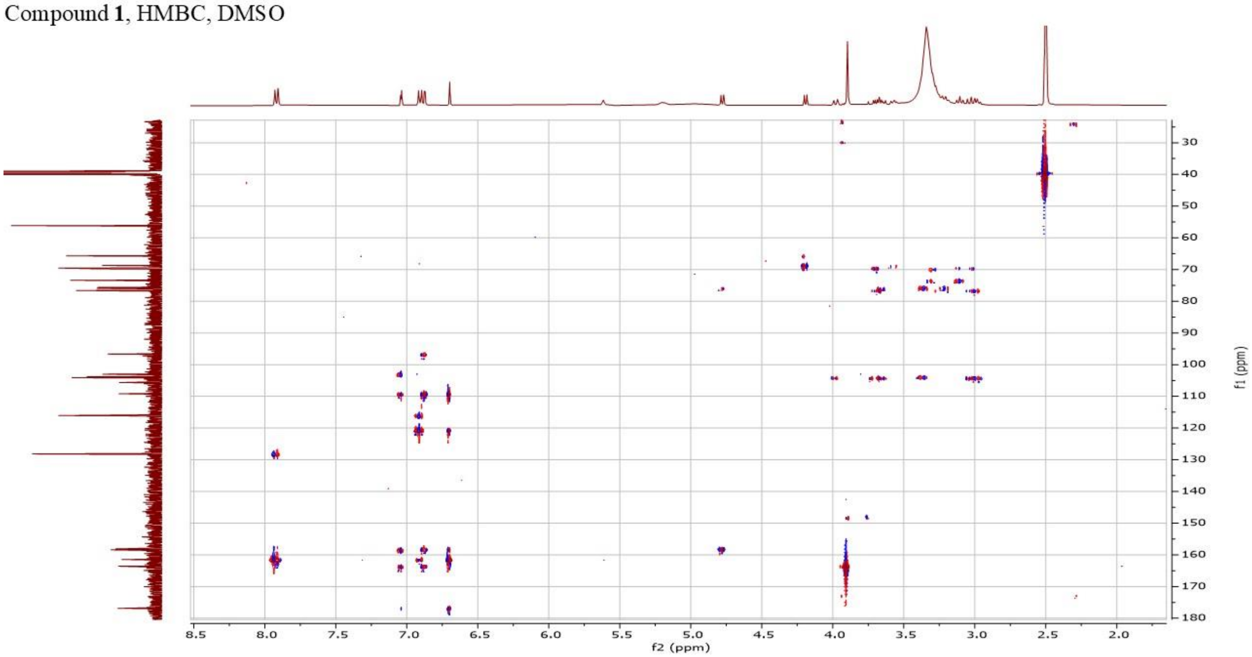


Fig.S 5. HMBC spectrum of compound **1**

Compound **1**, HH-TOCSY, DMSO

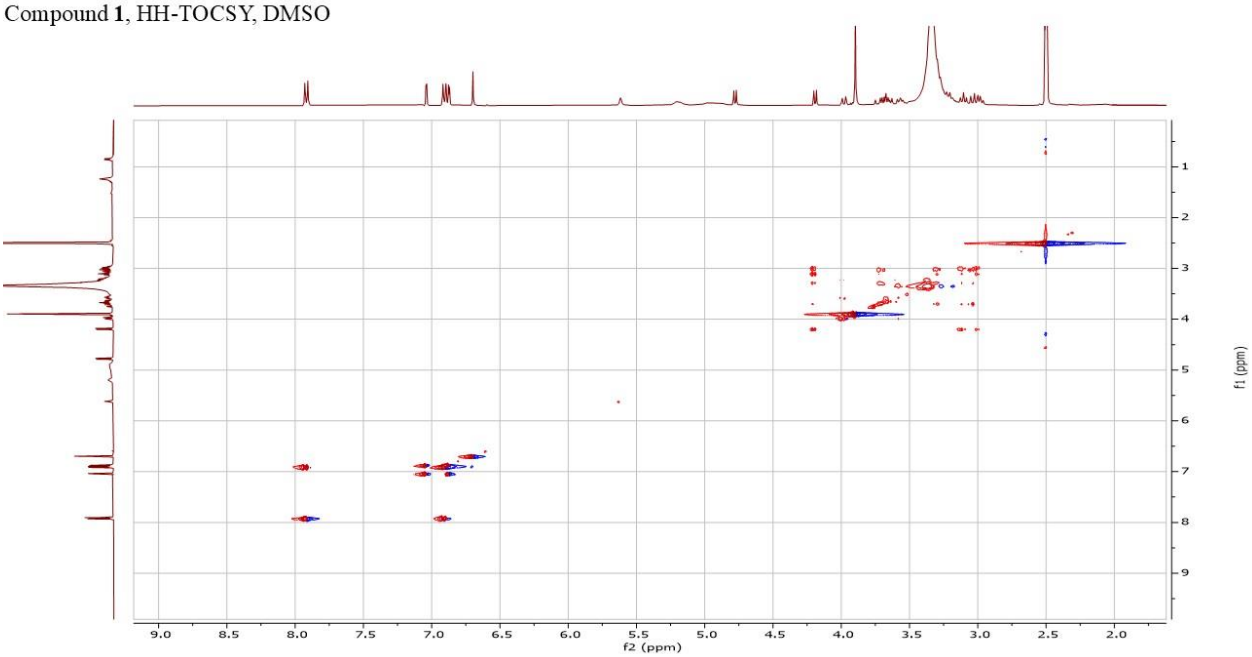

Fig.S 6.H-H TOCSY spectrum of compound **1**

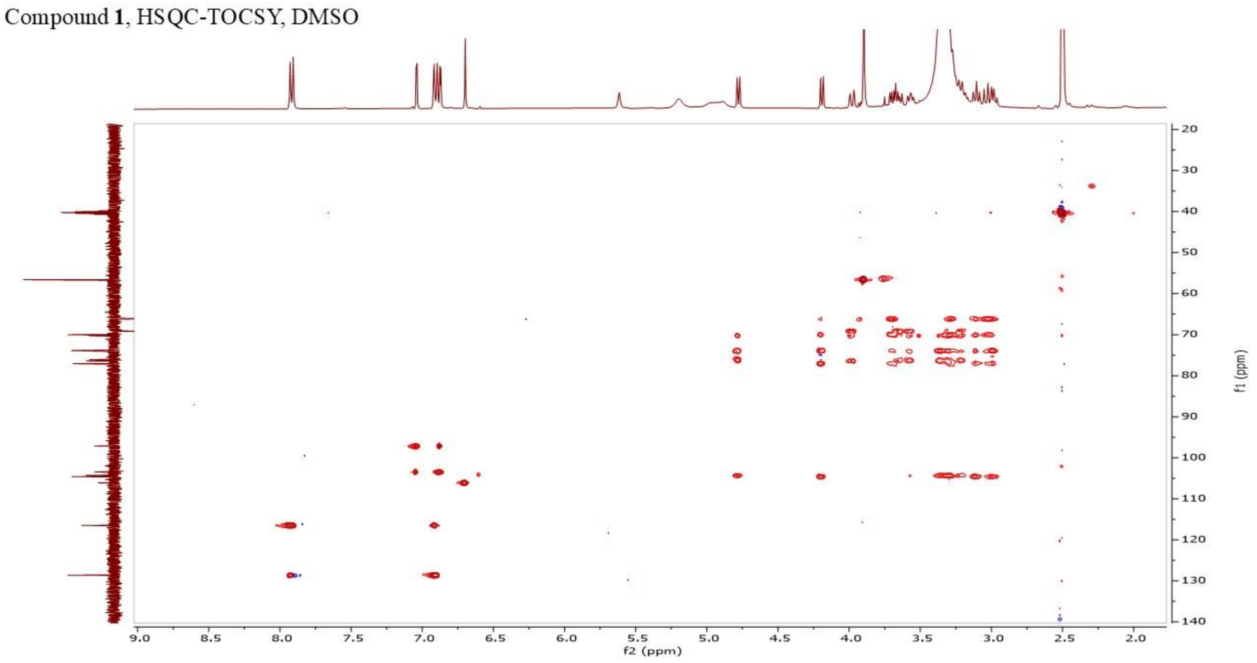


Fig.S 7. HSQC-TOCSY spectrum of compound **1**

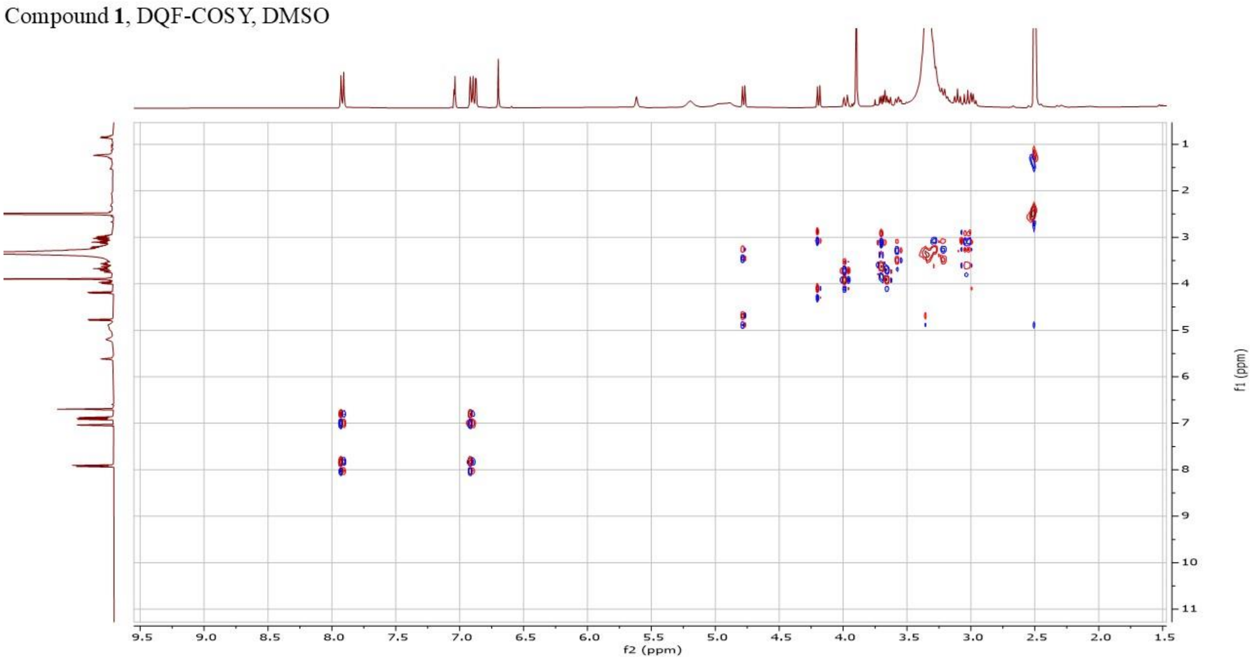

Fig.S 8. DQF-COSY spectrum of compound **1**

Compound **1**, exact MS spectrum

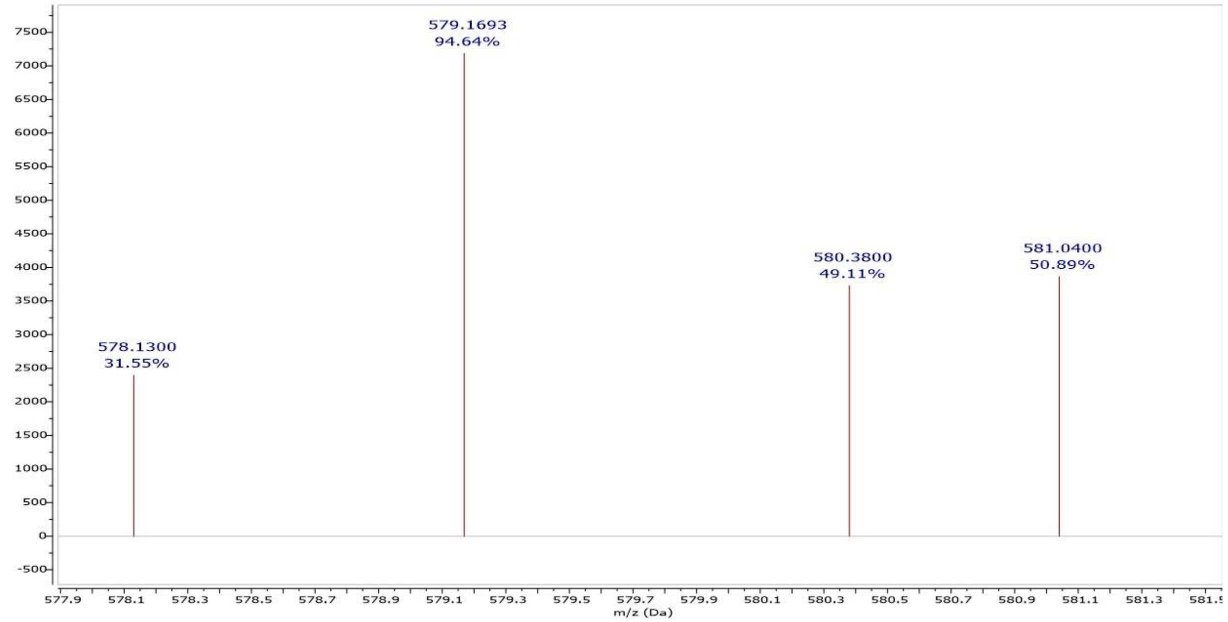


Fig.S 9. ESIMS spectrum of compound 1 m/z 579.1693[M - H]$^{-}$.

Compound **2**, H-NMR, DMSO

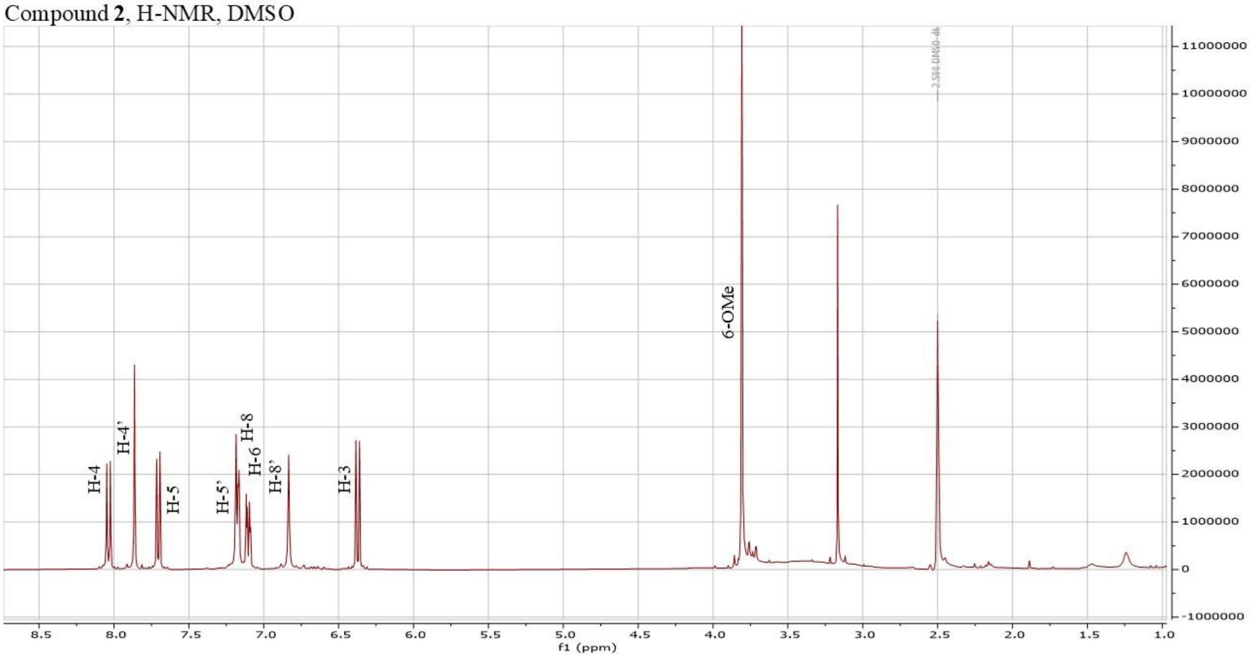

Fig.S 10.$^{1}$H NMR (400.15 MHz) spectrum of compound **2**

Compound **2**, C-NMR, DMSO

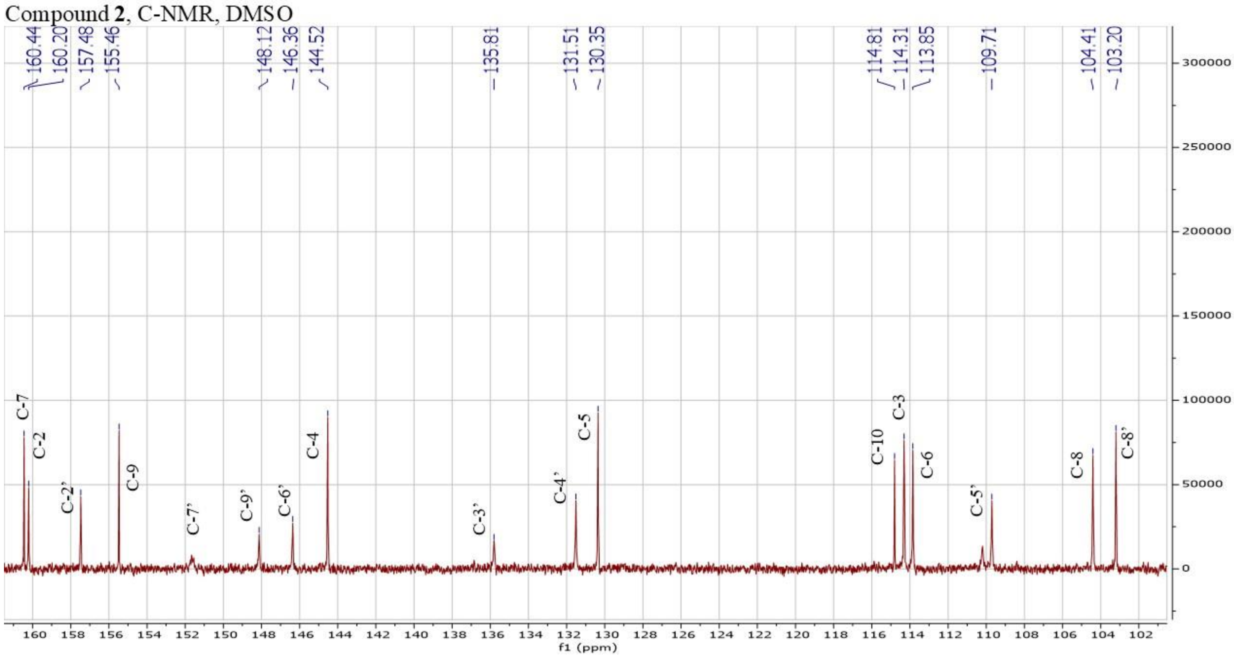


Fig.S 11.$^{13}$C NMR (100.6 MHz) spectrum of compound **2**

Compound **2**, DEPT-135, DMSO

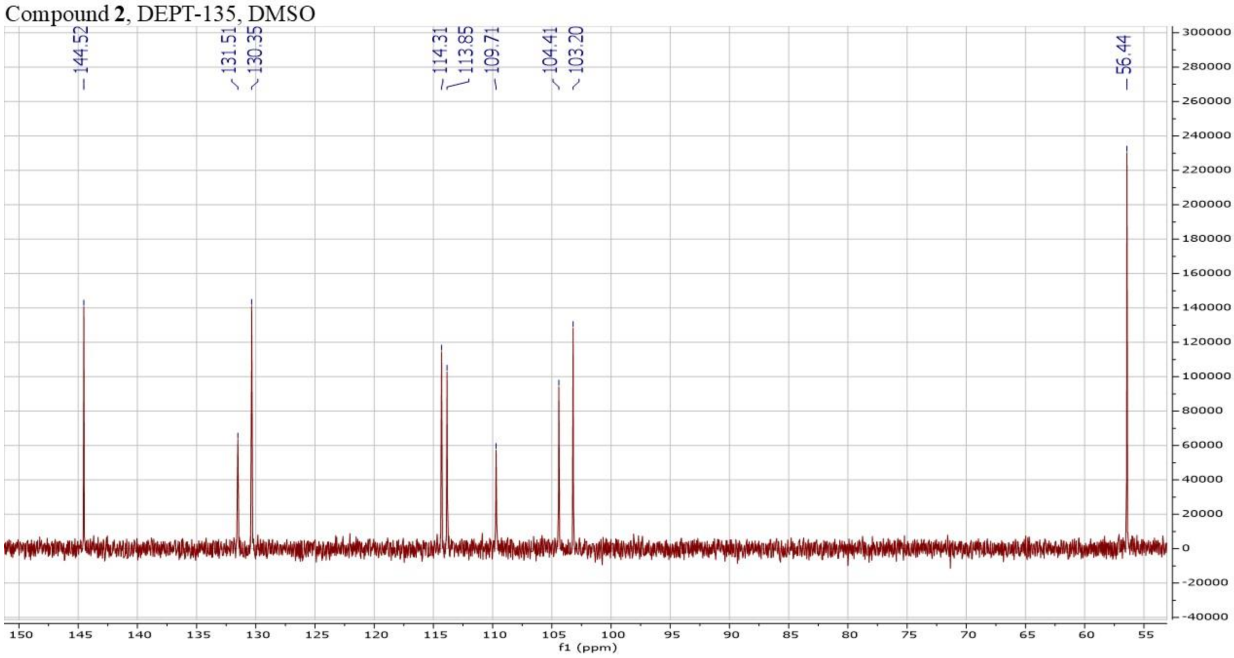

Fig.S 12. DEPT-135 spectrum of compound **2**

Compound **2**, HSQC, DMSO

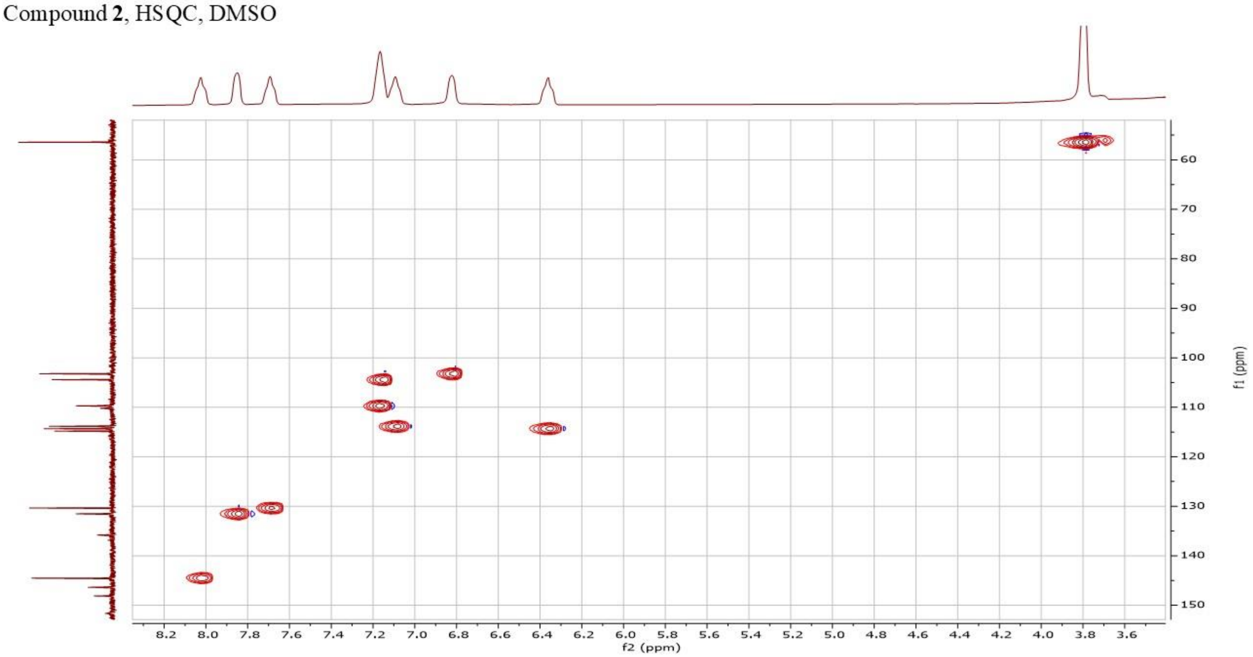

Fig.S 23.HSQC spectrum of compound **2**

Compound **2**, HMBC, DMSO

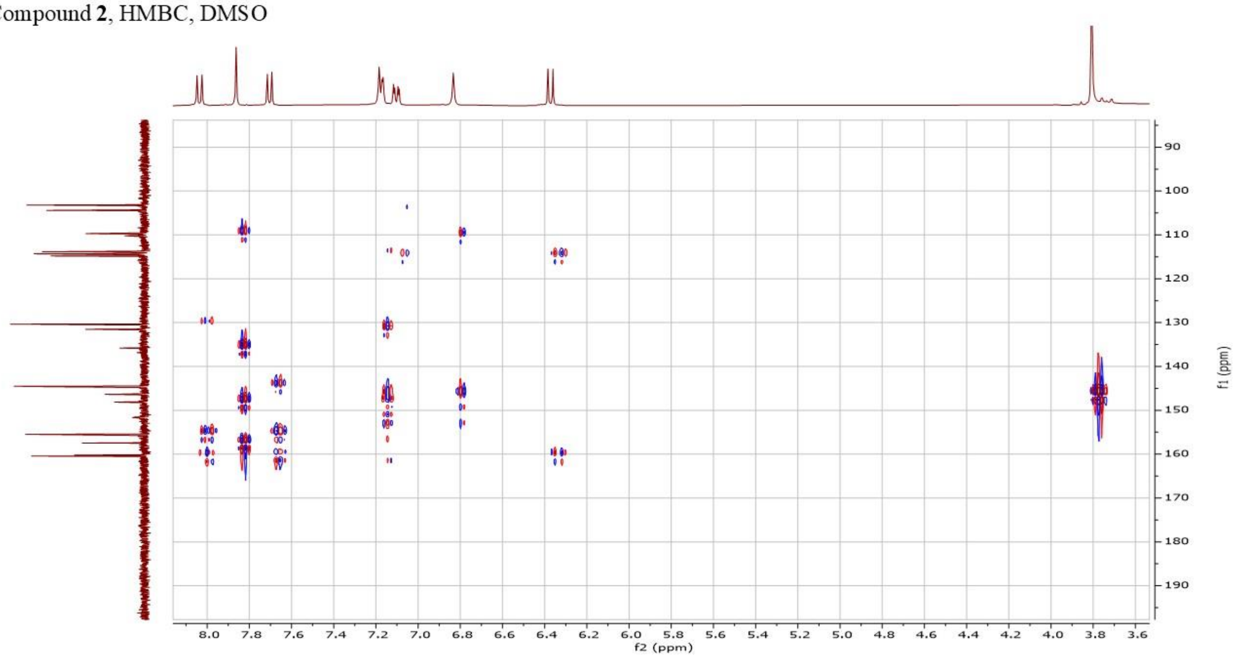

Fig.S 34.HMBC spectrum of compound **2**

Compound **2**, exact MS spectrum

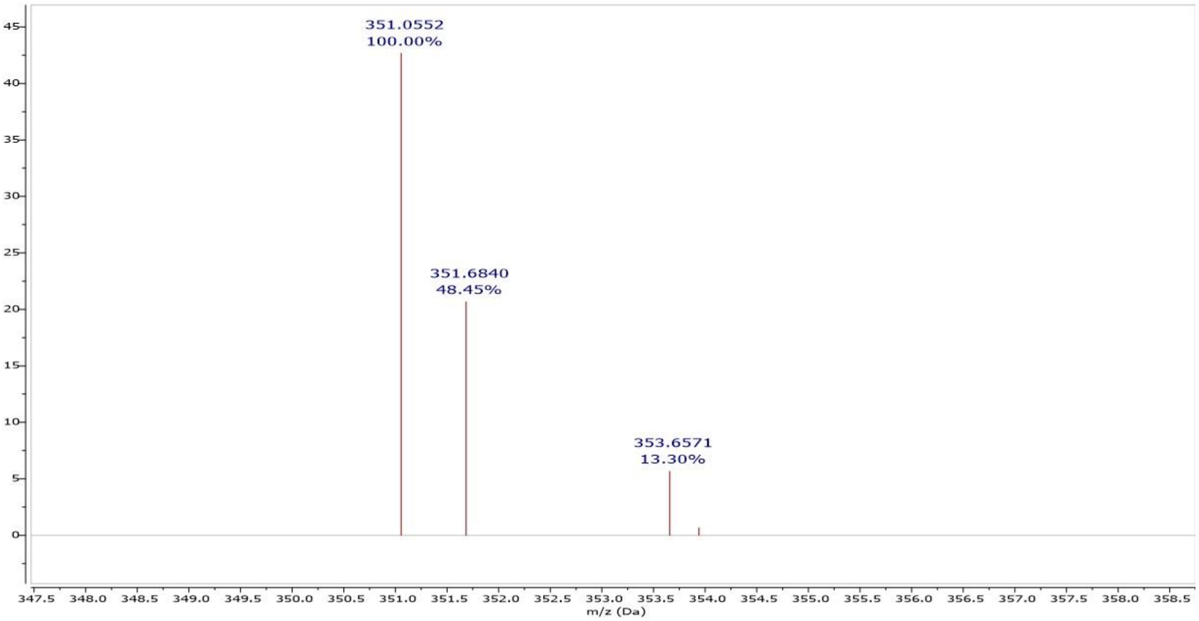


Fig.S 15. ESIMS spectrum of compound **2** m/z 351.0552[M - H]$^{-}$.

Compound **3**, H-NMR, CDCl3

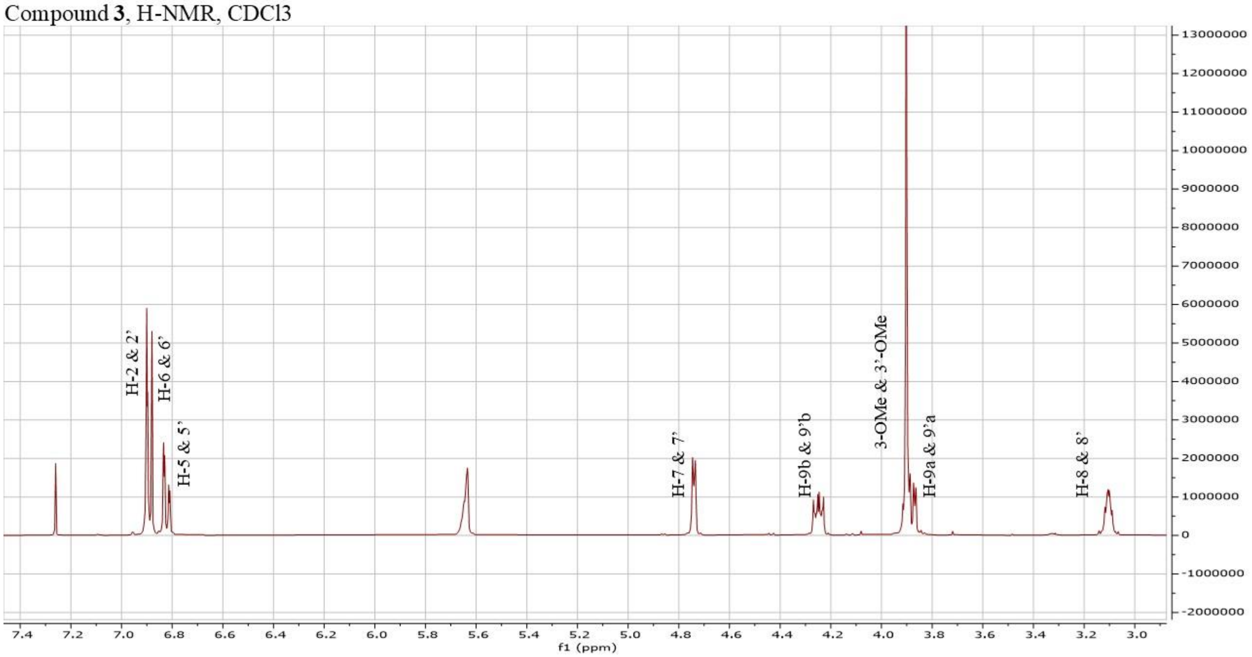


Fig.S 46.$^{1}$H NMR (400.15 MHz) spectrum of compound **3**

Compound **3**, C-NMR, CDCl3

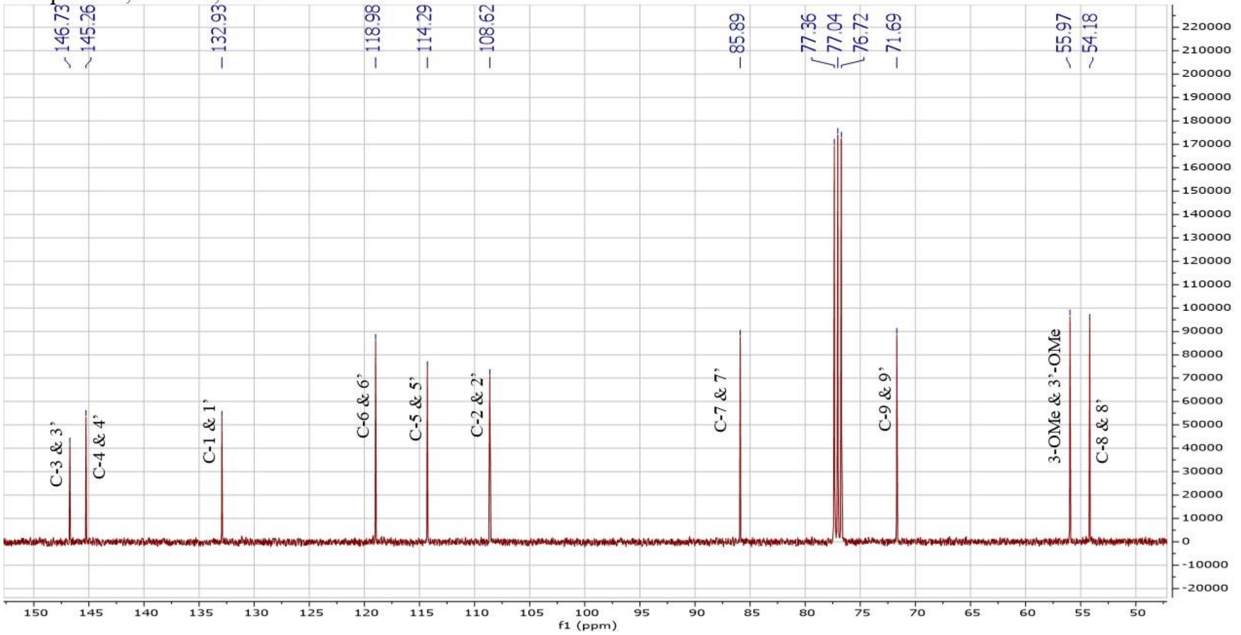


Fig.S 17.$^{13}$C NMR (100.6 MHz) spectrum of compound **3**

Compound **3**, DEPT-135, CDCl3

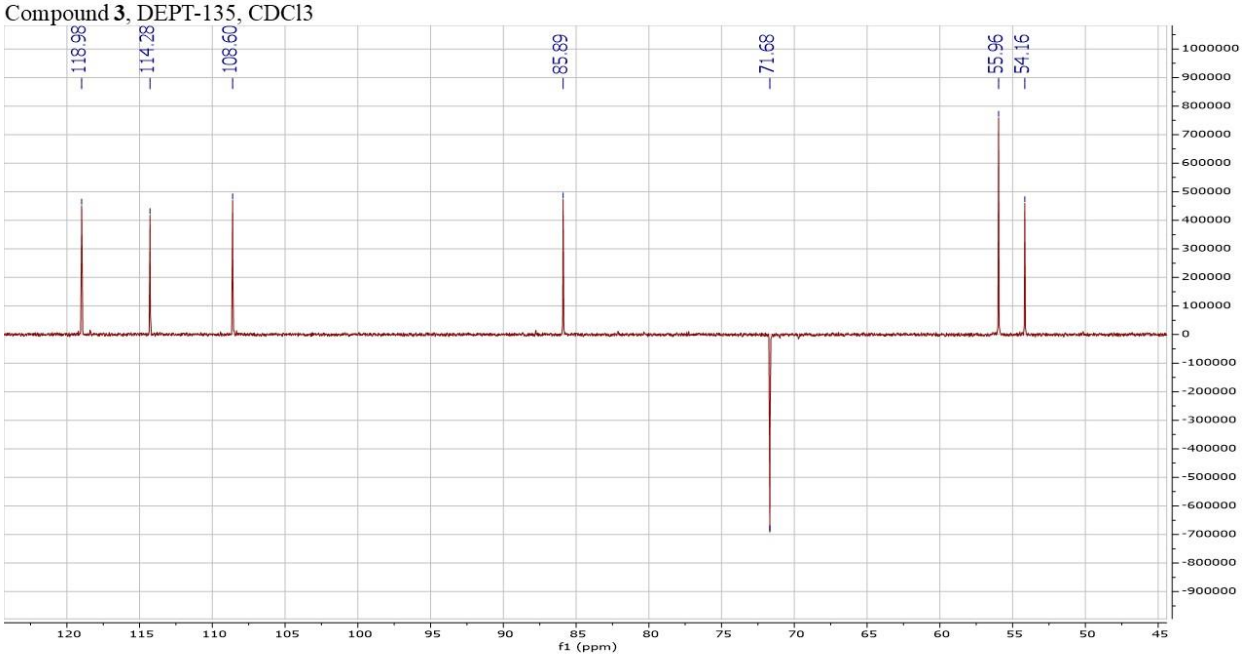

Fig.S 58.DEPT-135 spectrum of compound **3**

Compound **3**, DEPT-90, CDCl3

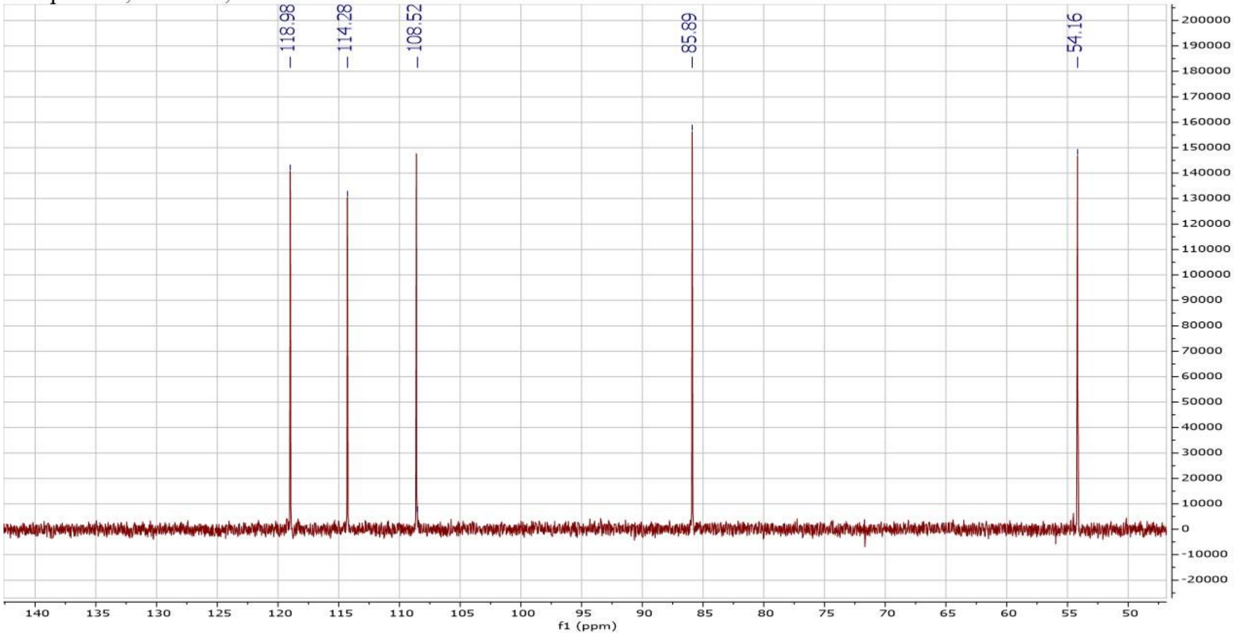


Fig.S 19. DEPT-90 spectrum of compound **3**

Compound **3**, HSQC, DMSO

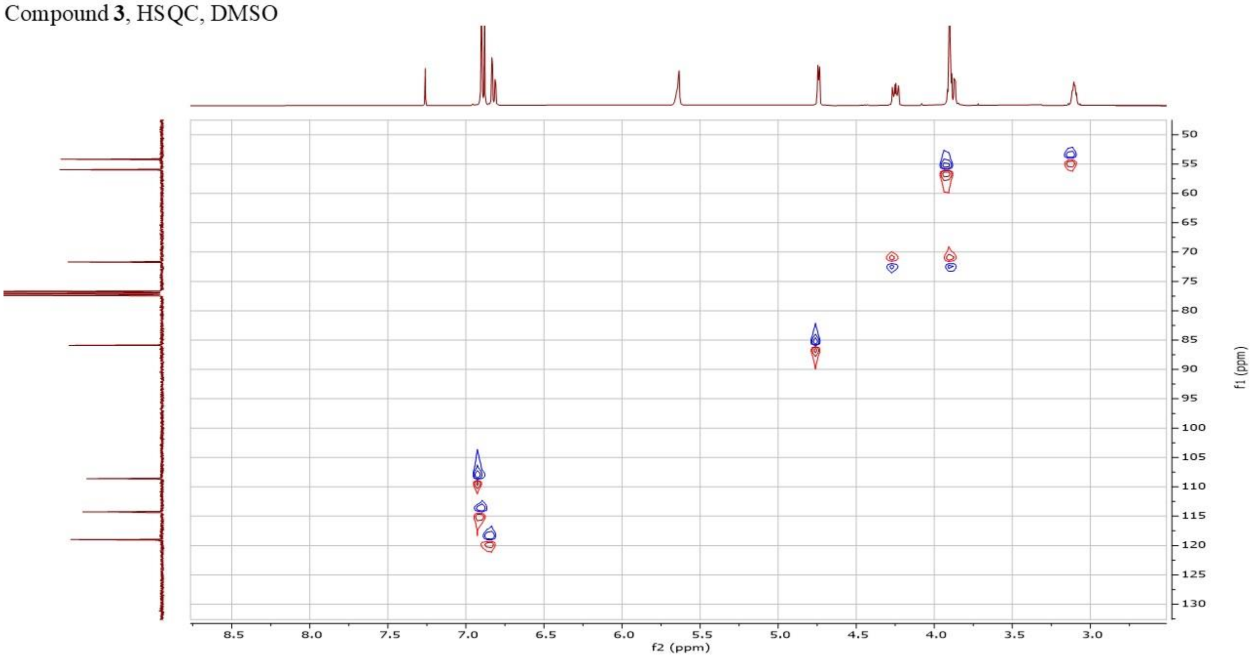

Fig.S 20. HSQC spectrum of compound **3**

Compound **3**, exact MS spectrum

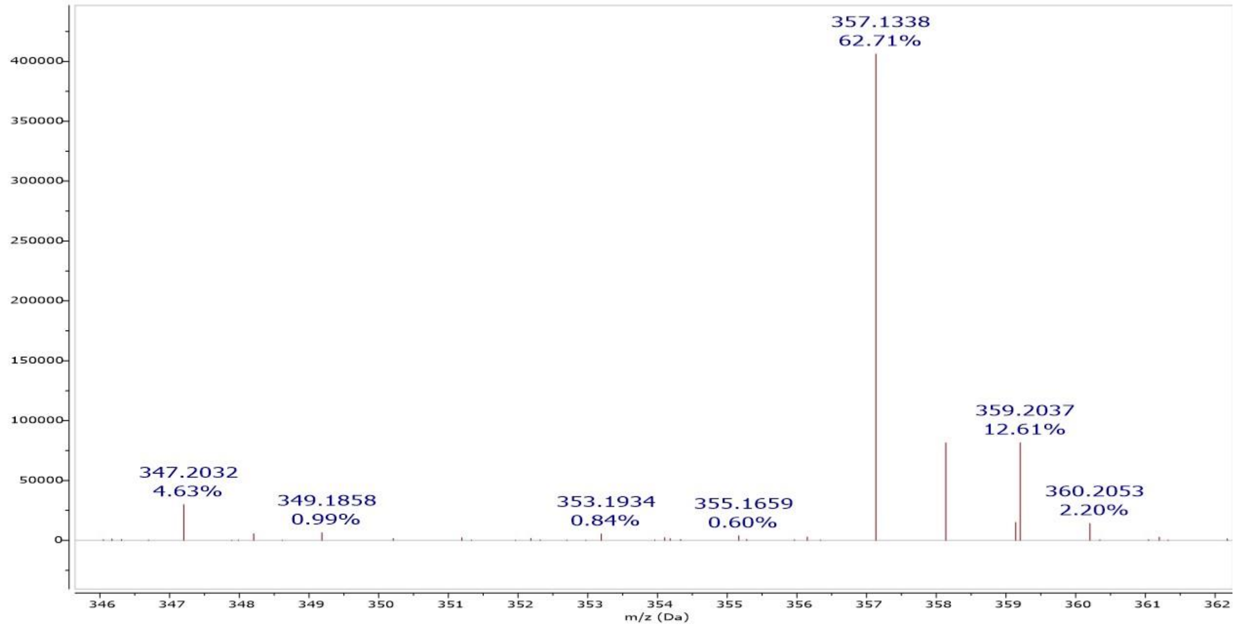


Fig.S 21. ESIMS spectrum of compound **3** m/z 357.1338[M - H] $^{-}$.
